# Whole genome sequencing analysis identifies sex differences of familial pattern contributing to phenotypic diversity in autism

**DOI:** 10.1186/s13073-024-01385-6

**Published:** 2024-09-27

**Authors:** Soo-Whee Kim, Hyeji Lee, Da Yea Song, Gang-Hee Lee, Jungeun Ji, Jung Woo Park, Jae Hyun Han, Jee Won Lee, Hee Jung Byun, Ji Hyun Son, Ye Rim Kim, Yoojeong Lee, Jaewon Kim, Ashish Jung, Junehawk Lee, Eunha Kim, So Hyun Kim, Jeong Ho Lee, F. Kyle Satterstrom, Santhosh Girirajan, Anders D. Børglum, Jakob Grove, Eunjoon Kim, Donna M. Werling, Hee Jeong Yoo, Joon-Yong An

**Affiliations:** 1 Department of Integrated Biomedical and Life Science, Korea University, Seoul, Republic of Korea; 2 L-HOPE Program for Community-Based Total Learning Health Systems, Korea University, Seoul, Republic of Korea; 3 Department of Psychiatry, Seoul National University Bundang Hospital, Seongnam, Republic of Korea; 4 Department of Psychiatry, Seoul National University College of Medicine, Seoul, Republic of Korea; 5 Korea Institute of Science and Technology Information, Daejeon, Republic of Korea; 6 Department of Psychiatry, College of Medicine, Soonchunhyang University Cheonan Hospital, Cheonan, Republic of Korea; 7 Department of Psychiatry, Soonchunhyang University College of Medicine, Cheonan, South Korea; 8 Department of Psychiatry, Seoul Metropolitan Children’s Hospital, Seoul, Republic of Korea; 9 School of Biosystem and Biomedical Science, College of Health Science, Korea University, Seoul, Republic of Korea; 10 School of Neuroscience, Korea University College of Medicine, Seoul, Republic of Korea; 11 BK21 Graduate Program, Department of Biomedical Sciences, Korea University College of Medicine, Seoul, Republic of Korea; 12 Department of Psychology, Korea University, Seoul, Republic of Korea; 13 Graduate School of Medical Science and Engineering, Korea Advanced Institute of Science and Technology, Daejeon, Republic of Korea; 14 Stanley Center for Psychiatric Research and Program in Medical and Population Genetics, Broad Institute of MIT and Harvard, Cambridge, MA USA; 15 Analytic and Translational Genetics Unit, Department of Medicine, Massachusetts General Hospital, Boston, MA USA; 16 Department of Biochemistry and Molecular Biology, Pennsylvania State University, University Park, PA USA; 17 The Lundbeck Foundation Initiative for Integrative Psychiatric Research, iPSYCH, Aarhus, Denmark; 18 Center for Genomics and Personalized Medicine, Aarhus, Denmark; 19 Department of Biomedicine, Aarhus University, Aarhus, Denmark; 20 Bioinformatics Research Centre, BiRC, Aarhus University, Aarhus, Denmark; 21 Department of Biological Sciences, Korea Advanced Institute of Science and Technology, Daejeon, Republic of Korea; 22 Synaptic Brain Dysfunctions, Institute for Basic Science, Daejeon, Republic of Korea; 23 Laboratory of Genetics, University of Wisconsin-Madison, Madison, WI USA

**Keywords:** Whole-genome sequencing, Autism, Sex difference, Phenotypic diversity, Familial pattern, Polygenic burden

## Abstract

**Background:**

Whole-genome sequencing (WGS) analyses have found higher genetic burden in autistic females compared to males, supporting higher liability threshold in females. However, genomic evidence of sex differences has been limited to European ancestry to date and little is known about how genetic variation leads to autism-related traits within families across sex.

**Methods:**

To address this gap, we present WGS data of Korean autism families (*n* = 2255) and a Korean general population sample (*n* = 2500), the largest WGS data of East Asian ancestry. We analyzed sex differences in genetic burden and compared with cohorts of European ancestry (*n* = 15,839). Further, with extensively collected family-wise Korean autism phenotype data (*n* = 3730), we investigated sex differences in phenotypic scores and gene-phenotype associations within family.

**Results:**

We observed robust female enrichment of de novo protein-truncating variants in autistic individuals across cohorts. However, sex differences in polygenic burden varied across cohorts and we found that the differential proportion of comorbid intellectual disability and severe autism symptoms mainly drove these variations. In siblings, males of autistic females exhibited the most severe social communication deficits. Female siblings exhibited lower phenotypic severity despite the higher polygenic burden than male siblings. Mothers also showed higher tolerance for polygenic burden than fathers, supporting higher liability threshold in females.

**Conclusions:**

Our findings indicate that genetic liability in autism is both sex- and phenotype-dependent, expanding the current understanding of autism’s genetic complexity. Our work further suggests that family-based assessments of sex differences can help unravel underlying sex-differential liability in autism.

**Supplementary Information:**

The online version contains supplementary material available at 10.1186/s13073-024-01385-6.

## Background

Autism exhibits a notable sex bias, with a 4:1 male-to-female prevalence [1–3]. This disparity suggests that females may have a higher genetic liability threshold for autism than males [4]. Large-scale whole-genome sequencing (WGS) analyses have found that females with autism have a greater incidence of de novo protein-truncating variants (PTVs) [5–7]. Moreover, the male-to-female ratio decreased to 3:1, in autistic children with intellectual disability (ID) [2, 8], a comorbid condition associated with de novo PTVs [6, 9, 10]. Recent studies also showed that in cases without ID, females have a higher polygenic score (PS) than males [11, 12]. These findings underscore the complexity of the genetic architecture of autism and its variable phenotypic variability and association across sexes.

Despite these advancements, the findings predominantly derive from populations of European ancestry, as seen in large WGS cohorts such as the Simons Simplex Collection (SSC), Simons Foundation Powering Autism Research (SPARK), and MSSNG [9, 13]. These cohorts are primarily composed of autism families of European ancestry and were recruited from the USA and Canada [13–15]. A WGS study from diverse populations would facilitate cross-ancestry comparisons as to genetic factors and phenotypic associations and enhance our understanding of sex differences in autism.

For a comprehensive study of sex bias in autism, two key aspects need to be considered. First, it is crucial to examine the core phenotypes of autism, as sex affects not only the presence of co-occurring intellectual disability but also the manifestations and measurements of core diagnostic features [16–18]. This deeper phenotypic characterization, especially for core autism symptoms, has not been extensively explored previously. Second, comparing different sexes among family members can provide a better spectrum of genetic factors and phenotypic expressions. Unaffected siblings and parents share a substantial genetic background with autism probands but have a lower chance of having autism-associated de novo variants. Analyzing family-wise phenotype datasets across sex would help investigate the inherited genetic influence on various clinical phenotypes across sex with better clarity.

In this study, we present the Korean autism family data, the largest data of East Asian ancestry, encompassing WGS data from 673 families of 2255 individuals and deep phenotyping data from 1499 families of 3730 individuals. We examined the genetic factors underlying sex differences in our cohort and compared with SSC and SPARK of European ancestry. Additionally, we performed a comprehensive phenotype analysis of sex differences, particularly in unaffected siblings and parents, and validated several findings in SSC and SPARK. Our findings suggest that comorbid ID and total severity of autism core symptoms modulate sex differences in de novo and polygenic burden and provide evidence of sex-differential liability threshold for diverse autism-associated clinical features within families.

## Methods

### Cohorts comprising Korean autism data

Data for this cohort, comprising of individuals with autism and their families, was collected from three major hospital sites in Korea: Seoul National University Bundang Hospital (SNUBH) served as the primary center, overseeing the study at Soon Chun Hyang University Hospital Bucheon (SCHBC) and Seoul Child Hospital (SCH). All recruited families were approved by the ethics committee of SNUBH, SCHBC, and SCH Institutional Review Boards (IRB) (SNUBH: B-1703–388-303 and B-2108–700-107; SCHBC: SCHBC 2018–04-020 and SCHBC 2022–04-016; SCH: P01-201908-BM-02 and P01-202111–21-003). This study adhered to the ethical standards of the Helsinki Declaration and informed consent was obtained from all study participants. We aimed to recruit participants with diverse clinical features and to achieve a balanced sex ratio among individuals with autism.

### A note on terminology

In this paper, we used the term “individuals with autism”, “autistic individuals”, or “autism cases/probands” for individuals clinically diagnosed with autism, according to Rolland et al. 2023 [19]. We used the term “non-autistic siblings and parents” to refer to siblings and parents of individuals with autism, who do not have clinical features which meet the diagnostic criteria. For the general population, we used the term “control population.” We must acknowledge that several of these individuals in the general population may also present with autism.

### Samples

For the Korean autism cohort, we collected data from Korean families with at least one child diagnosed with autism by clinicians. We collected DNA samples from whole blood and clinical phenotypes from participants. All phenotype information was cross validated by clinical specialists. The collected data were fully anonymized and handled in accordance with the biorepository’s standard operating procedures. A total of 1400 families (*n*_individuals_ = 3730) were collected and their clinical data were used for phenotype analysis. Of these, WGS data was generated for 673 families, including 696 individuals with autism, 213 non-autistic siblings and 1346 parents (Additional file 2: Table S1) used for de novo and polygenic score analysis. Compared with the previous release [20], 39 families were newly added in the Korean autism WGS dataset. Of the 673 families, 21 (3.1%) were multiplex families with more than two autistic children.

For the Korean general population WGS data, we accessed the Korean Genome and Epidemiology Study (KoGES; The National Project of Bio Big Data) genomic resource. The KoGES study had collected data from clinically non-diagnosed adults, aged > 40 years from Ansan and Ansung [21]. We downloaded a joint VCF file and clinical data from The National Project of Bio Big Data (www.nih.go.kr/biobank/). A total of 2500 participants (1272 female and 1228 male individuals) were used for polygenic score analysis in this study (Additional file 2: Table S1).

For the SSC, and SPARK cohorts, we downloaded a joint VCF file and clinical data from SFARI Base (https://sfari.org/sfari-base). For the SSC cohort [14], we excluded twin and ancillary collection and employed only the simplex collection. We used total of 1855 families of European ancestry (*n*_individuals_ = 6976) for genetic burden test and all fully phasable 4318 trios for de novo gene discovery analysis from the WGS data (v2019-05–12). We used phenotype data (v15.3) from a total of 2644 families (*n*_individuals_ = 10,456), for subsequent phenotype analysis (Additional file 2: Table S1). Similarly, for the SPARK cohort [15], we used total of 2434 families of European ancestry (*n*_individuals_ = 8863) for genetic burden test and all fully phasable 5683 trios for de novo gene discovery analysis from the WGS data (v1.1). We additionally utilized fully phasable 25,325 trios for de novo gene discovery analysis from SPARK WES data (v2). For phenotype analysis, 108,266 families (*n*_individuals_ = 149,547) from SPARK phenotype data (v9) were used (Additional file 2: Table S1).

### Sequencing data

For Korean autism cohort, DNA was obtained from whole blood of the subjects and sequenced on Illumina Hiseq X at sequencing read depth 30x. We processed WGS data using the Illumina DRAGEN germline pipelines (v4.0.3) [22], and variant calling for the human reference genome version GRCh38. Multi-sample joint genotyping was conducted using iterative gVCF genotyper.

For KoGES, DNA was extracted from whole blood of subjects and sequenced on Illumina NovaSeq 6000 with an average of 30 × read depth. Sequencing reads were aligned to human reference genome GRCh38. Subsequent processing followed GATK Best Practices (v4.2.4.1). After joint genotyping of individual gVCF, variant quality scores were recalibrated by VQSR.

For SSC, DNA was obtained from whole blood of the subjects and sequenced on Illumina Hiseq X10. Sequencing reads were aligned to human reference genome GRCh38. Subsequent processing of the alignments followed GATK Best Practices (v3.5). After joint genotyping of individual gVCF, variant quality scores were recalibrated by VQSR and low-quality genotypes (GQ < 20; DP < 10) were converted to missing genotypes. Only variants with “PASS” entries in the FILTER column were used for the downstream analysis. For SPARK, DNA was obtained from saliva of the subjects and prepared with PCR-free methods and sequenced on Illumina NovaSeq 6000. Sequencing reads were aligned to human reference genome GRCh38. Subsequent processing of the alignments followed GATK Best Practices (v3.5). Joint genotyping of individual gVCF was conducted by GLnexus (v1.4.1).

### Quality control for samples and variants

Quality control (QC) for samples and high-quality (HQ) variants was conducted by Hail 0.2 (https://hail.is/) and Peddy [23]. For sample QC, we checked the distribution of SNPs, INDELs, transition/transversion (ti/tv) ratio, genotype quality, and genotype depth in a sample level to see if there are outliers. We also calculated relatedness between individuals in a dataset and imputed biological sex and ancestry. For Korean autism and KoGES datasets, all samples passed the sample QC (Korean autism *n* = 2255; KoGES = 2500). For SSC and SPARK datasets, we excluded sex/pedigree mismatched samples and used only European ancestry.

For Korean autism dataset, we retained variants labelled as “PASS” in the DRAGEN hard-filter and excluded those occurring within low complexity regions (LCR). Additionally, multiallelic sites were split into biallelic sites. Prior to this, local allele expressions, including allele depth (AD) and localized phred-scaled genotype likelihood (LPL), were transformed into a global format. For homozygous reference calls, AD were substituted with an array filled with read depth (DP) for the reference alleles, with zeros for other alleles, and LPL were replaced with “NA.” Localized alternative alleles (LAA) were converted to local alleles array (LA) by adding zero to the first element of LAA. Furthermore, the maximum LPL value was added to the empty elements of LPL to convert them into PL. Following the multi-allele split, PL values for homozygous reference calls, initially marked as “NA,” were annotated with an array consisting of 0, genotype quality (GQ), and DP multiplied by 3. We also removed variants with allele length ≥ 50.

For detecting HQ rare variants, quality metrics in the whole filtering pipeline were optimized, according to the previously established practice [24]. We filtered variant calls with following cutoffs; heterozygous SNPs with QUAL ≥ 7.5, GQmean ≥ 36, and DPmean ≥ 34, 0.275 ≥ allele balance (AB) ≥ 0.725; heterozygous indels with QUAL ≥ 10.51, gDP ≥ 3, 0.214 ≥ AB ≥ 0.786; homozygous alternative SNVs with QUAL ≥ 20.3, AN ≥ 4312, AB ≥ 0.905, GQmean ≥ 15.7, DPmean ≥ 12, GQ ≥ 9, gDP ≥ 11; homozygous alternative indels with QUAL ≥ 24.78, AN ≥ 3504, GQmean ≥ 29, DPmean ≥ 11.55, GQ ≥ 1, gDP ≥ 5, AB ≥ 0.905. We further filtered variant calls with call rate < 10% and a Hardy–Weinberg equilibrium *P* < 1 × 10^−12^.

For detecting HQ common variants, we filtered variant calls using following criteria; GQ ≥ 20, DP ≥ 10, AB 0.2–0.8 for heterozygous calls and AB ≥ 0.95 for homozygous calls, call rate ≥ 95%, Hardy–Weinberg equilibrium *P* ≥ 1 × 10^−6^. We then utilized variants with internal unrelated AF more than 0.05.

For SSC and SPARK dataset, we excluded variants in LCR, split multi-allelic sites, and removed INDELs with allele length ≥ 50. To filter HQ common variants, we used the same filtering pipeline with Korean autism dataset. For SSC, we applied further filtering for HQ rare variants with QC metric cutoffs, referring to the previous work [24].

### Identification of de novo variants

De novo variants (DNVs) were called by the Hail built-in de_novo() function in the annotated variants with the internal allele frequency (AF) less than 0.001 and gnomAD (v3.1) AF less than 0.001 in the non-psychiatric disease subset. We used the default cutoffs of Hail de_novo() function for further filtering: AB_parent_ ≤ 0.1, AB_child_ < 0.3, DP_child_/(sum of DP_parents_) ≥ 0.3 and GQ ≥ 20.

For the Korean autism data, we modified the method to calculate the de novo probability [25] in Hail de_novo() function considering the partial origin in which DNV occurred. The modified approach calculates the probability that a DNV has occurred, together with the probability that it was inherited from parents. We obtained the de novo probability, for each of the following five conditions:

- DNV was from mother. … a
- DNV was from father. … b
- The genotype of the mother was not homozygote, and the alternative allele was inherited. … c
- The genotype of the father was not homozygote, and the alternative allele was inherited. … d
- The genotype of the child was not heterozygote. … e

Among the probabilities obtained, the ratio of the probability that the variant represents a true DNV (referred to as the de novo probability) was calculated as follows:

$$De\ novo\ probability=\frac{a+b}{a+b+c+d+e}$$

Another consideration was the GQ scale of DRAGEN. The DRAGEN uses an algorithm that can reduce errors in actual data where correlations between reads are observed [22]. Therefore, DRAGEN genotypes have lower distribution of GQ than GATK. As such, we tried to adjust the threshold of de novo probability to rescue false-negative calls whose confidence was low due to the lower GQ distribution. To determine the optimal cutoff, we measured the number of obtained DNVs lowering the threshold from 0.5 to 0.05 and set the de novo probability threshold to 0.1. We further filtered DNVs found in less than five individual cases. This step identified 47,269 autosomal DNVs in individuals with autism (*n* = 696) and 14,309 in non-autistic siblings (*n* = 213) (Additional file 3: Table S2). Consistent with the previous genetic studies on autism [26–29], there was a positive correlation between paternal age and the number of DNVs for each sample (0.13 DNVs per paternal age month, *P* < 2.2 × 10^−16^) (Additional file 1: Fig.S1A).

For the SSC and SPARK data, we filtered high/medium confidence DNVs using the original cutoffs for the de novo probability. We excluded samples that presented with excessive number of DNVs due to pedigree errors. We filtered DNVs with internal AC = 1, unless the DNVs were identified in monozygotic twins. For SPARK, we further filtered DNVs with AB < 0.8. In the SSC data, we identified 119,785 autosomal DNVs in autism cases (*n* = 1838) and 95,874 in non-autistic siblings (*n* = 1504). In SPARK, we identified 167,623 autosomal DNVs in individuals with autism (*n* = 2532) and 103,861 in non-autistic siblings (*n* = 1591). We observed a positive correlation between paternal age and the number of DNVs per sample in both datasets (SSC – 0.13 DNVs per paternal age month, *P* < 2.2 × 10^−16^; SPARK – 0.13 DNVs per paternal age month, *P* < 2.2 × 10^−16^) (Additional file 1: Fig. S1A).

### Variant annotation

HQ variants were annotated with Hail vep() function with Ensembl variant effect predictor (VEP) version 109.3. With the most severe consequence term annotated by VEP, we classified variants into three categories, PTV, missense variant (MIS), and synonymous. PTV included the “frameshift_variant,” “splice_acceptor_variant,” “splice_donor_variant,” and “stop_gain” variants with high confidence by LOFTEE plugin [30] with no LOFTEE flags other than “SINGLE_EXON.” The “missense_variant,” “stop_lost,” “start_lost,” and “protein_altering_variant” were labelled as missense. Lastly, we defined the “synonymous_variant,” “stop_retained_variant,” and “incomplete_terminal_codon_variant” as synonymous.

### Computation of PS

Using HQ common variants, we calculated the PS for autism. For autism, we used two European-ancestry Genome Wide Association Study (GWAS) summary statistics, one from Grove et al. [31], which includes SSC and iPSYCH data (*n* = 46,350), and the other which includes only the iPSYCH dataset (unpublished) (*n* = 58,948). We used the former base data for calculating PS in the Korean and SPARK cohorts and the latter for calculating PS in SSC, as the overlaps between the target data and the base data could inflate the polygenic signal.

To match the SNP format between the input data used for PS calculation, we conducted SNP matching for the target data and GWAS summary statistics. Prior to SNP matching, we carried over the target data from GRCh38 to GRCh37 to match the genome build of GWAS summary statistics. Then SNP matching was conducted as described below. We united the allele representation as “1st to 13th nucleotide + (length of allele – 13)” when the allele was longer than 13 bp. The INDELs which localized in the same locus but were reverse of each other, as well as ambiguous SNPs, {“A”, “T”}, {“T”, “A”}, {“C”, “G”}, {“G”, “C”}, were excluded. We then matched those with the SNPs list from the linkage disequilibrium (LD) reference comprised of HapMap3 SNPs from UKBB European individuals, provided from PRScs [32]. We used the European LD reference as it more closely matched the ancestry with the GWAS summary statistics. For target data which had different ancestry other than European, Korean autism, and KoGES, we harmonized AF by the chi-square test. If the AF difference of one SNP between the target and the reference was significantly different from the mean by more than 1 SD of all matched SNPs, the SNP was excluded.

We computed the PS using four different calculation methods: PRScs [32], SBayesR [33], LDpred2 [34], and PRSice [35]. The PRScs, SBayesR, and LDpred2 calculate PS by implementing Bayesian shrinkage of beta effect size of SNPs weighed by LD, whereas PRSice calculates PS by using several SNPs that pass the optimal *P-*value cutoffs. Given that the adjustment for polygenic risk using PRScs improved the prediction the most [36], we used PRScs as the primary calculation tool. Parallel computation of 22 autosomes was performed with default parameter: gamma distribution for local shrinkage (1, 0.5) and phi value for global shrinkage 1.0 × 10^−2^. The results of PS were consistent across four different methodologies (Additional file 1: Fig. S2). The correlation coefficients were especially high between shrinkage methods (PRScs, SBayesR, and LDpred2), on average 0.8, but between shrinkage methods and *P*-value cutoff method the correlations were lower than that, 0.6.

### Sex-specific gene analysis

To identify autism-associated genes, we ran transmitted and de novo association gene discovery (TADA) analysis, Bayesian association algorithm [6, 37]. We used de novo PTVs and damaging MIS in all genes from full phasable trios with autistic child in Korean WGS (*n* = 696), SSC WGS (*n* = 2380), SPARK WGS (*n* = 3496), and WES (not overlapped with WGS samples; *n* = 17,473). We performed TADA in females (total 4885) and males (total 19,160) respectively and identified female genes and male genes. Next, we conducted Gene Ontology set enrichment test and visualized network of enriched pathways using clusterProfiler package (v4.11.1) in R.

### Clinical phenotype data

To investigate sex differences in clinical features, we assessed core symptoms including total symptom severity (summed score of social communication deficits and restricted/repetitive behaviors), social communication deficits, and restricted/repetitive behaviors and cognitive/adaptive function. Higher phenotypic scores of autism core symptoms reflect a clinical outcome with more distinct features of autism, whereas lower phenotypic scores of cognitive/adaptive functions indicate more impaired cognitive/adaptive ability.

### Total symptom severity

Overall severity of autism-related symptoms was assessed using a variety of instruments. The Autism Diagnostic Observation Schedule-2 (ADOS-2) [38] was administered to children and their siblings, utilizing different modules tailored to each participant’s age and verbal language ability. For comparison across the different modules, the total calibrated severity scores (CSS) were used for analysis. For the Korean autism cohorts, we used the Korean-translated version [39] of ADOS-2 that was approved by the Western Psychological Services. Additionally, caregivers completed the social responsiveness scale (SRS) [40] and the Social Communication Questionnaire (SCQ) [41, 42], which measure the overall severity of autistic symptoms. For the SRS, T-scores were used, while for the SCQ, scores from both the current and lifetime versions were included in the analysis. Parents also completed self-reported questionnaires, including the Autism Quotient [43] and the broad autism phenotype questionnaire (BAPQ) [44]. Across all instruments, higher scores were indicative of greater severity in autism-related symptoms.

### Social communication deficits

To evaluate social communication skills, social affect CSS scores from the ADOS-2 (ADOS SA) were utilized, alongside the autism diagnostic interview, revised (ADIR) [41] social interaction (ADIR A) and communication domains (ADIR B). The ADI-R, consisted of a semi-structured interview with caregivers, was administered by trained professionals who rated each question item. Based on the diagnostic algorithm, four domain scores were aggregated. Considering each participant’s developmental trajectory, the communication domain within the ADI-R was further divided into subdomains, specifically catering to individuals with and without fluent verbal communication (ADIR B verbal and non-verbal). Consistent with the scoring approach of the ADOS-2 CSS, higher scores on the ADI-R indicated greater difficulties in social communication.

### Restricted interest and repetitive behavior

Restricted interest and repetitive behaviors (RRB) were measured using both the RRB CSS from the ADOS-2 and the RRB subdomain of the ADI-R (ADIR C).

### Cognitive ability

Intelligence was assessed using the Wechsler Preschool and Primary Scale of Intelligence (WPPSI) [45], Wechsler Intelligence Scale for Children (WISC) [46], and Wechsler Adult Intelligence Scale. For individuals with limited verbal language abilities, the Leiter international Performance Scale-Revised (non-verbal IQ) [47] was administered to measure non-verbal IQ.

### Adaptive behaviors

The Vineland adaptive behavior scale (VABS)-II [48] was utilized to assess adaptive functioning in children and their siblings. The VABS-II is a caregiver-report questionnaire covering various domains, with each item inquiring whether the child can perform the specified task. Four domain scores—socialization, communication, daily living, and motor skills—along with a composite score, were calculated and standardized based on age-matched normative control individuals. Lower scores in each of these domains were indicative of developmental delays.

### Statistical analyses

For the DNV association tests, we prioritized DNVs with loss-of-function observed over expected upper bound fraction (LOEUF) score [30] for PTV and missense badness, PolyPhen-2, and constraint (MPC) score [49] for MIS. The de novo PTV with LOEUF < 0.37, and MIS with MPC ≥ 2 were used for association test. We estimated the power in DNV association test for de novo PTV and MIS in Korean autism cohort and compared the result with that from a previous report [7] in more than 20,000 samples in European-ancestry autism cohorts. Although we lacked the statistical significance threshold during the DNV analysis for the Korean autism cohort, power estimation revealed that this was likely due to a limited sample size (Additional file 1: Fig. S3; Additional file 3: Table S2). The burden of de novo PTV and MIS was compared between individuals with autism and non-autistic siblings, and between autistic females and males, using a one-sided binomial test. For comparing the percentage of samples with de novo PTVs between individuals with autism ± ID, and siblings, we used the Fisher’s exact test.

For polygenic burden association tests, we compared the PS across groups and sexes, using two-sample *t* tests. To assess the relative difference of polygenic burden, we compared the OR and *P*-value from the logistic regression as follows:

Group (cases with certain phenotype severity versus siblings) ~ PS.

We compared clinical phenotypes across groups and sexes, using two-sample *t* tests and two-way ANOVA tests, followed by Tukey’s test for multiple comparisons. To ensure the sex differences in clinical phenotypes in cases, siblings, and parents, we adjusted *P-*values from two-sample *t* tests with the number of clinical features for each domain.

## Results

### Overview of autism family data of East Asian and European ancestry

**Fig. 1:**
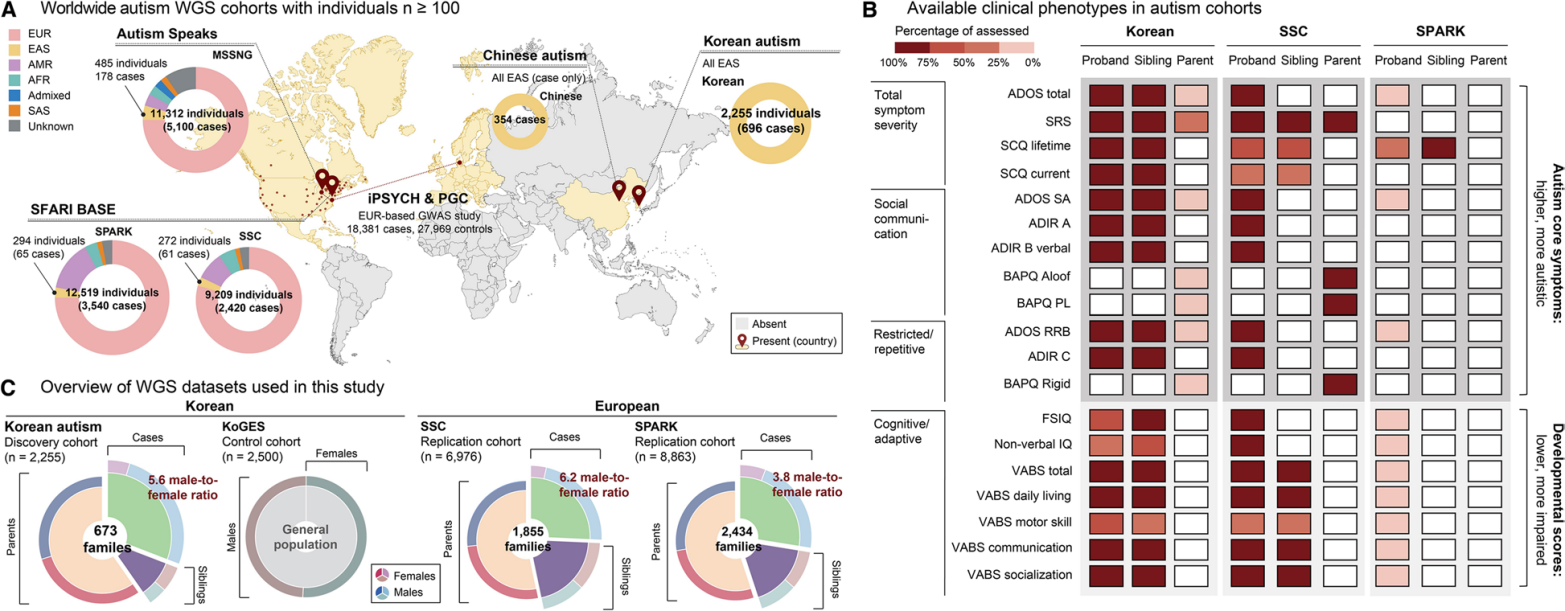
Overview of WGS and phenotype datasets used in this study. **A** Worldwide distribution of autism WGS cohorts with individuals ≥ 100. The largest published autism GWAS data, to date, is also illustrated in the map. The size of each cohort is illustrated by the size of bands and the composition of ancestries are represented by colors in bands (peach, European; yellow, East Asian; lavender, American; turquoise, African; blue, Admixed; orange, South Asian; gray, Unknown ancestry). Red points mark the location of each consortia/cohort and colored areas further delineate the geographic breadth of participant recruitment. Created with BioRender.com. **B** Comparison of assessable phenotypic scores in autism families in Korean, SSC, SPARK data. Phenotypic scores investigated consist of autism core symptoms including total symptom severity, social communication deficits, and restricted/repetitive behaviors (higher, more autistic) and developmental scores including cognitive/adaptive scores (lower, more impaired). The percentage of phenotypes assessed in each cohort are represented by shades of red (redder, higher coverage). **C** Overview of the WGS datasets used in this study. Composition of samples, including groups and sexes, is displayed with pie plot. Male-to-female ratio in children with autism is depicted in red letters. For replication cohorts, we subset samples with European ancestry. Groups are represented by colors in inner rings (green, autism cases; purple, non-autistic siblings; apricot, parents) and sexes are represented by colors in outer rings (pink, female cases; light blue, male cases; dark pink, female siblings; blue-green, male siblings; red, mothers; blue, fathers)

The Korean autism WGS data consists of 673 families of total 2255 individuals, encompassing 21 multiplex families with more than two autistic children. The Korean autism WGS data is the largest autism WGS data of East Asian ancestry (Fig. 1A). This data set outnumbers not only the published Chinese autism cohort (354 individuals) [50] but also includes a greater number of individuals of East Asian ancestry than are represented within major global autism WGS datasets including SSC (272 individuals) [14], SPARK [15] (294 individuals), and MSSNG [13] (485 individuals) (Fig. 1A). The Korean autism cohort extensively collected deep phenotype data from these 673 families and an additional 826 families of 1475 individuals (total 1499 families of 3730 individuals) (Fig. 1B). In this paper, we categorized 19 phenotypes into three different domains including (1) total symptom severity (summed score of core autism features [51, 52]—social communication deficits and restricted/repetitive behaviors), (2) social communication deficits, (3) restricted/repetitive behaviors, and development-associated features including cognitive/adaptive function domain.

We compared the number and the coverage of phenotype collected in Korean autism cohort with the SSC (2644 families, 10,456 individuals) and SPARK (108,266 families, 149,547 individuals) cohorts (Fig. 1B) (Additional file 2: Table S1). The number and depth of each phenotype assessed in the Korean autism cohort were comparable to SSC and higher than SPARK. Of note, 16 different features were measured for siblings and eight for parents in the Korean dataset, which was twice as rich as the SSC dataset, and many of these measures were absent in the SPARK dataset.

The Korean autism WGS data includes 696 children with autism, 213 non-autistic siblings and 1346 parents (Fig. 1C) (Additional file 2: Table S1). We found a high male to female ratio in children with autism, but this was not observed in siblings, consistent with previous reports [2, 3]. Of the 696 individuals with autism, 590 were males and 106 were females (5.6 male-to-female ratio). Siblings included 90 male and 123 female individuals (0.74 male-to-female ratio). For replication cohorts, we analyzed individuals of European ancestry from the WGS data of SSC [14] (1855 families, 6976 individuals) and SPARK initiative [15] (2434 families, 8863 individuals). Both datasets included a greater number of males with autism than females with autism (male-to-female ratio 6.2 in SSC; 3.8 in SPARK) (Fig. 1C).

### Sex differences of autism-associated genetic burden

**Fig. 2:**
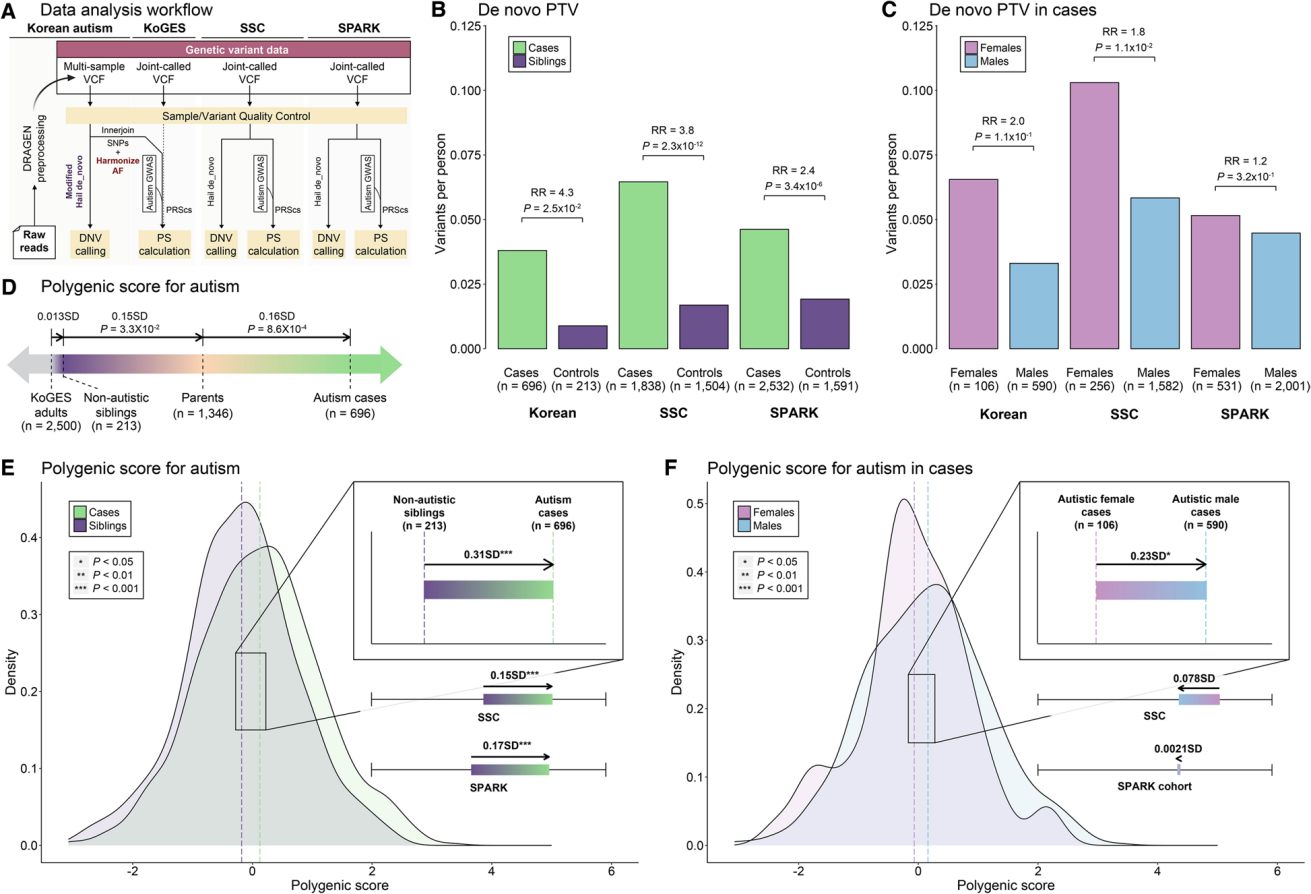
Autism-associated de novo and polygenic burden and their sex differences. **A **Data analysis workflow. DNV calling and PS calculations were conducted separately for each cohort, with the exception of the Korean autism and KoGES datasets, where SNP intersection and PS calculations were carried out jointly. For the Korean autism and the KoGES cohort, AF harmonization was performed to align with European-derived associations. For Korean autism data, preprocessing of raw reads, and multi-sample genotyping were conducted by DRAGEN. **B**, **C** Comparison of the de novo PTVs in constrained genes (LOEUF < 0.37), adjusted for paternal age at birth across the Korean autism, SSC, and SPARK cohorts; **B** between children with autism and non-autistic siblings; **C** between sex among children with autism. The y axis indicates the average number of variants. The *P*-values were computed by one-sided exact binomial test. Groups and sexes are represented by colors (green, autism cases; purple, non-autistic siblings; pink, female cases; light blue, male cases). **D** The Korean continuum of polygenic burden for autism in the general population and families with autism cases. Group differences are standardized using the distribution of PS in KoGES samples and *P*-values were computed by two-sample t tests. Deviations and *P*-values are depicted for only the nearest group comparisons within a continuum of polygenic score. Groups are represented by colors (green, autism cases; purple, non-autistic siblings; apricot, parents; gray, KoGES adults). **E**, **F** The distribution of PS; E in children with autism and non-autistic siblings; **F** in female and male cases in Korean autism families. Dashed lines and colored bar in zoomed area correspond to mean PS of each group in Korean families, and colored bars under the zoom-in box correspond to group differences of PS in SSC and SPARK. The direction of arrows represents the direction of enrichment **E** from cases to non-autistic siblings and F from females to males. Group differences are standardized using the distribution of PS in families and *P*-values were computed by two-sample t tests. Significance level is denoted by asterisk (“***”, *P* < 0.001; “**”, *P* < 0.01; “*”, *P* < 0.05). Groups and sexes are represented by colors (green, autism cases; purple, non-autistic siblings; pink, female cases; light blue, male cases)

We conducted a quality control assessment for the WGS datasets as per previous WGS studies [24, 53, 54] and prioritized high-quality variants for de novo and common variant analyses (Fig. 2A). We restricted de novo analysis to PTVs in genes of LOEUF scores [30] < 0.37 genes and MIS with MPC scores [49] ≥ 2. Our WGS analyses revealed a higher rate of de novo PTVs and MIS in children with autism compared to non-autistic siblings (Fig. 2B;Additional file 1: Fig. S1; Additional file 3: Table S2). Consistent with existing findings [6, 7, 55, 56], de novo PTVs were found to be significantly enriched in children with autism in both Korean and replication cohorts (Fig. 2B). In children with autism, de novo PTVs were observed more frequently in females than males in Korean cohort (RR = 2.0; 0.066 variants per female and 0.033 per male; *P* = 0.11), SSC (RR = 1.8; 0.104 per female case and 0.058 per male case; *P* = 1.1 × 10^−2^), and SPARK (RR = 1.2; 0.052 per female case and 0.045 per male case; *P* = 0.32) (Fig. 2C; Additional file 4: Table S3). Although the difference was only significant in SSC, these results are consistent with a higher liability threshold in autistic females than males.

We further aimed to identify autism-associated genes in autistic females and males separately according to the previous framework with de novo variants [6, 37] (Additional file 1: Fig. S4A) (Additional file 5: Table S4). We identified 98 autism-associated genes in females and 461 genes in males, of which 58 genes overlapped. Identified genes were enriched for biological pathways associated with regulation of chromatin regulation, histone modification, synaptic functions, and cytoskeleton, in line with the previous study [6, 55] (Additional file 1: Fig. S4B). Female-specific genes were enriched for chromatin regulation and histone modification, whereas male-specific genes were highly observed in synaptic functions. Nonetheless, none of the pathway groups was exclusively affected by female- or male-specific genes.

Next, we calculated the PS for autism using the recent GWAS data of European ancestry [31]. To compare the polygenic burden with the general population, we utilized the Korean Genome and Epidemiology Study (KoGES; The National Project of Bio Big Data) dataset, which consists of WGS for 2500 Korean adults (Fig. 1C). To minimize potential bias caused by different ancestry, we harmonized allele frequency of those SNPs with European LD reference (Fig. 2A). European-derived PS performed similarly in Korean as in European ancestry [9, 11, 31, 57], with autistic children showing significantly higher PS than KoGES adults (0.32 SD; *P* = 3.74 × 10^−13^) and also siblings (0.31 SD; *P* = 5.2 × 10^−5^) (Fig. 2D, E; Additional file 3: Table S2). Unlike de novo PTVs, the PS enrichment for the two sexes was not consistent across the Korean, SSC and SPARK cohorts. While male children with autism showed significantly higher PS than female children in the Korean cohort (0.23 SD; *P* = 2.3 × 10^−2^), the opposite pattern was observed in SSC (0.078 SD; *P* = 0.24), and no sex bias was found in SPARK (0.002 SD; *P* = 0.97) (Fig. 2F; Additional file 4: Table S3).

### Intellectual disability and total symptom severity affect sex differences in genetic burden

While a higher burden of de novo PTVs in autistic females than autistic males were consistently observed in the Korean, SSC, and SPARK cohorts, the SPARK cases exhibited less pronounced female enrichment of de novo PTVs compared to the Korean and SSC cohorts. For polygenic burden, the three cohorts showed different patterns of sex-biased enrichment among individuals with autism. To further investigate the sex differences in genetic burden in the Korean, SSC, and SPARK cohorts, we compared the clinical phenotypes associated with de novo and polygenic burden in autistic females and males.

**Fig. 3:**
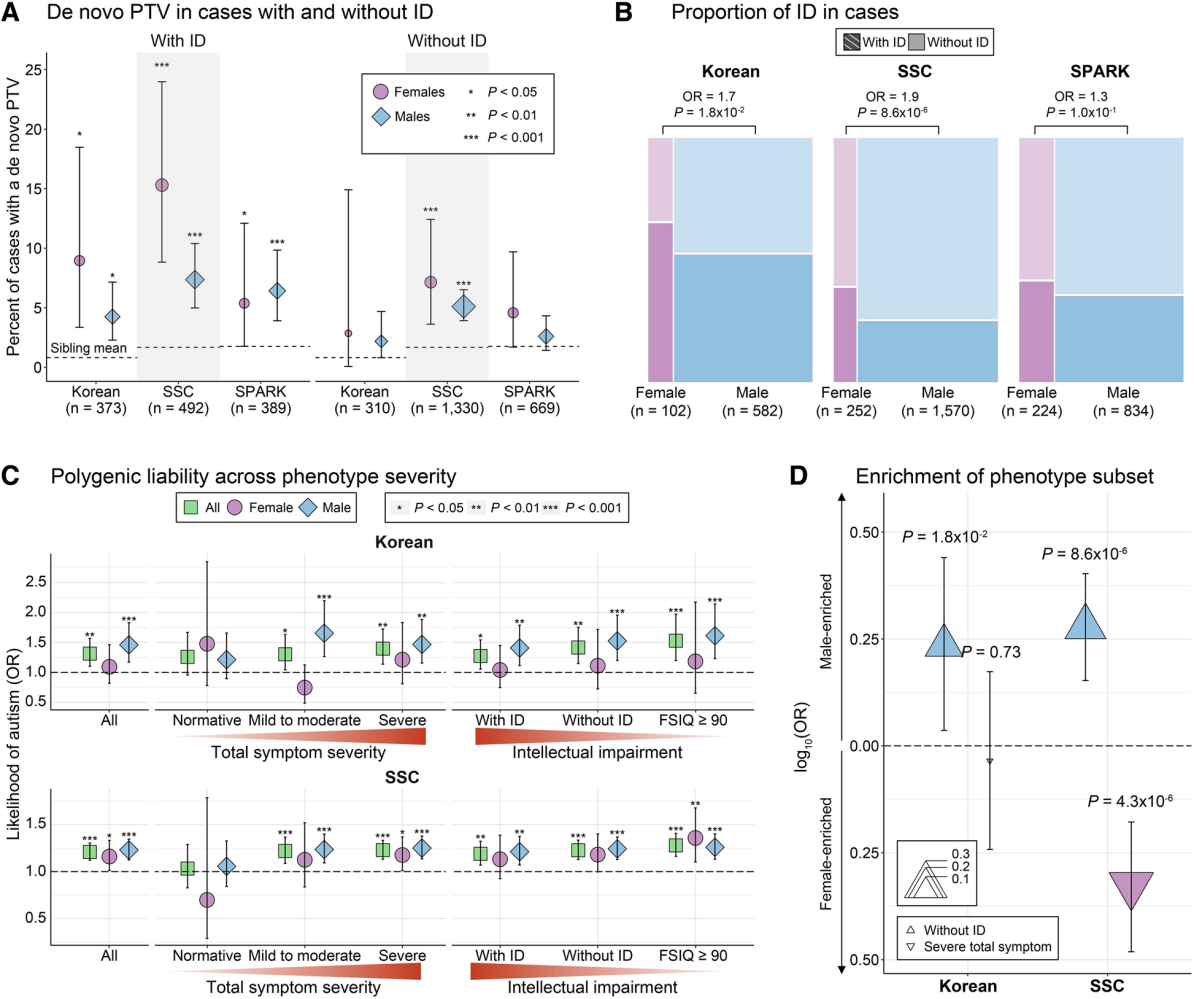
Effects of ID and total symptom severity on sex differences in de novo and polygenic burden. **A **The percent of autism cases carrying a de novo PTV in females and males, with or without ID. The *P* -values were calculated using a one-sided Fisher’s exact test and 95% confidence intervals (CIs) were calculated using a binomial test with siblings. Dashed line displays the percent of siblings carrying a de novo PTV. Significance level is denoted by asterisk (“***”, *P*  < 0.001; “**”, *P*  < 0.01; “*”, *P*  < 0.05). Sex is represented by colors and shapes (light blue circle, female cases; pink diamond, male cases). **B **Proportion of individuals with and without ID among autistic females and autistic males. The OR and *P*- values were calculated using two-sided Fisher’s exact test. Sex is represented by colors (pink, female cases; light blue, male cases) and co-occurring ID state is displayed by patterns (autism with ID, slashed; autism without ID, no pattern). **C **The relative difference of PS depending on the degree of total symptom severity of autism and comorbid intellectual impairments between individuals with autism and siblings. The OR and *P*- values were calculated using a logistic regression. Error bars represent the 95% CIs of OR. Significance level is denoted by asterisk (“***”, *P*  < 0.001; “**”, *P*  < 0.01; “*”, *P*  < 0.05). Sex is represented by colors and shapes (green rectangle, total cases; light blue circle, female cases; pink diamond, male cases). **D **Enrichment of phenotype subset, autistic individuals without comorbid ID and with high total symptom severity, across sexes. The OR and *P*- values were calculated using two-sided Fisher’s exact test. The direction of enrichment is represented by colors (pink, female-enriched; light blue, male-enriched), phenotype subset is displayed by shapes (up-pointing triangle, without ID; down-pointing triangle, high total symptom severity), and the magnitude of enrichment is represented by the size of triangle

The presence of de novo PTVs has been reported to be associated with lower cognitive and adaptive function, measured by full-scale and non-verbal IQ, and VABS [6, 9, 10]. Using these scores, we stratified cases into those with and without ID. In line with previous findings [58], we observed an average twofold higher proportion of individuals with a de novo PTV among autism cases with ID compared to those without ID in both females and males (Fig. 3A; Additional file 6: Table S5). The incidence of ID was higher in female cases than in male cases in all three cohorts (Fig. 3B; Additional file 6: Table S5). However, the relative difference in ID co-occurrence between sexes was smaller in SPARK (OR = 1.29, 95% CI = 0.94–1.76) than in the Korean (OR = 1.72, 95% CI = 1.09–2.76) and SSC (OR = 1.90, 95% CI = 1.42–2.52) cohort, which supported the less prominent enrichment of de novo PTVs among females with autism in SPARK.

Recent studies have observed that high polygenic score is associated with high SRS [9, 12, 59] and lower chance of comorbid ID. The SRS is a summed score for social communication deficits and restricted/repetitive behaviors which is commonly used as a clinical measure for core symptom severity of autism [40, 60]. We assessed the relative difference of polygenic burden across the degree of total severity of autism core symptoms, measured by SRS (normative SRS < 60; mild to moderate SRS 60–75; severe SRS > 75) [61], and comorbid intellectual impairments. We found polygenic burden in cases relative to siblings increases when total symptom severity increases (normative, OR = 1.26, 95% CI = 0.97–1.67; mild to moderate, OR = 1.30, 95% CI = 1.05–1.63; severe, OR = 1.40, 95% CI = 1.14–1.73) in the Korean cohort (Fig. 3C; Additional file 6: Table S5). On the other hand, polygenic burden in cases relative to siblings decreased as comorbid intellectual impairments increased (i.e., as IQ decreased; FSIQ ≥ 90, OR = 1.53, 95% CI = 1.20–1.97; without ID, OR = 1.42, 95% CI 1.15–1.75; ID, OR = 1.27, 95% CI = 1.05–1.54) in the Korean cohort (Fig. 3C; Additional file 6: Table S5). These results were consistently observed in SSC. We also observed significant positive correlations of PS with SRS and FSIQ (Additional file 6: Table S5), consistent with previous reports [9, 12, 59].

While both the Korean and SSC cohorts exhibited an association of polygenic burden with higher total symptom severity and lower likelihood of co-occurring ID, there were different distributions of total symptom severity of autism and comorbid ID in females and males. The Korean cohort had significantly higher proportion of autistic individuals without ID in males than females but the proportion of cases with high total symptom severity was not significantly different across sexes (Fig. 3D; Additional file 6: Table S5). While the SSC cohort also had significantly higher proportion of autistic individuals without ID in males than females, the proportion of cases with high total symptom severity was significantly higher in females, which accounts for the reversed sex differences compared to the Korean cohort (Fig. 3D; Additional file 6: Table S5). Further, there were no significant sex differences in PS when correcting for SRS and IQ (Additional file 6: Table S5). This finding suggests that variations in comorbid ID and total symptom severity drive varying sex differences of PS across cohorts.

### Intellectual disability and total symptom severity in male-to-female autism prevalence

**Fig. 4:**
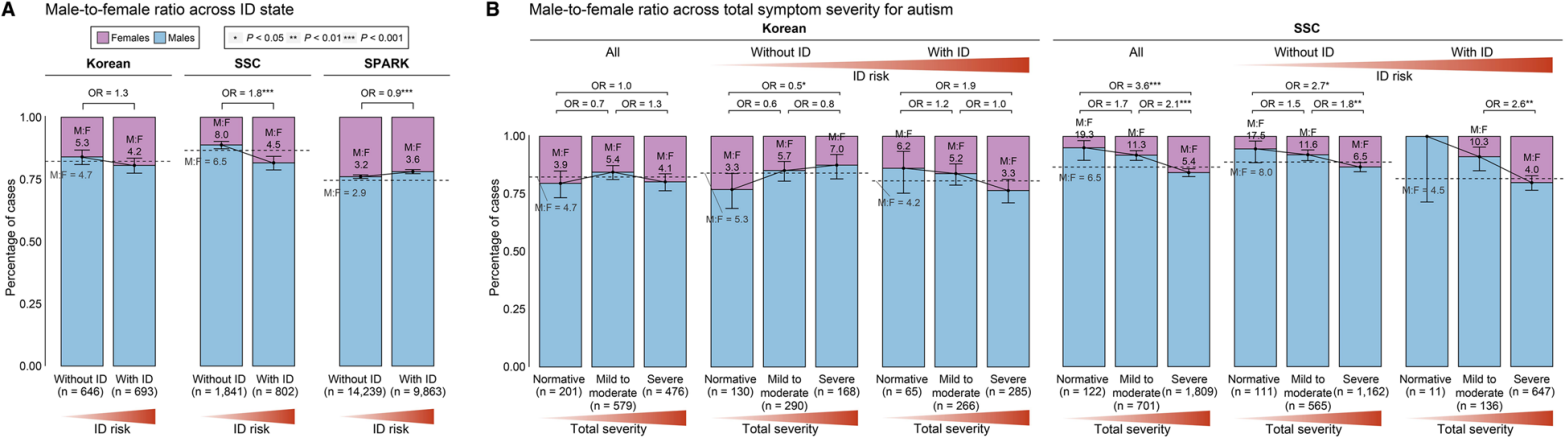
Male-to-female sex ratio in children with autism depending on comorbid ID and total symptom severity. **A**,**B** The number of females and males and male-to-female ratio; **A** in children with autism across ID comorbid state; **B** in children with autism depending on total symptom severity and ID comorbid state. The y axis and bar plot represent the percentage of female and male samples. Dashed lines correspond to the sex ratio in children with autism of each group (total cases, cases without ID, or cases with ID). The *P* -values were calculated using two-sided Fisher’s exact test across phenotype subgroups and 95% CIs were computed by two-sided exact binomial test with base male-to-female ratio in each subgroup in each cohort. Sex and groups are represented by colors of stacked bar plot (pink, female cases; light blue, male cases). Phenotypic severity is displayed by shades of red (redder, more severe)

Associations between de novo PTVs and PS with comorbid ID and total symptom severity of autism, support the relationship between genetic liability and phenotypic severity. Considering these correlations together with the sex-differential liability threshold, we may expect that as the phenotype’s severity increases (autism with comorbid ID or high total symptom severity), the male-to-female sex ratio will decrease [8, 62, 63] (Additional file 1: Fig. S5). As per hypothesis, we observed lower male-to-female sex ratio in autistic individuals with comorbid ID than those without comorbid ID across two of our three cohorts (Fig. 4A; Additional file 7: Table S6). In Korean cohort, the male-to-female ratio was 5.3 in autistic individuals without comorbid ID, and 4.2 in autistic individuals with ID (OR = 1.26; 95% CI = 0.94–1.70). Similarly, a decrease in the sex ratio was observed in the SSC cohort (8.0 for autism without ID; 4.5 for autism with ID; OR = 1.79; 95% CI = 1.41–2.27), whereas a marginal increase in the sex ratio was noted for the SPARK cohort (3.2 for autism without ID; 3.6 for autism with ID; OR = 0.89; 95% CI = 0.84–0.95).

When examining the male-to-female ratio based on total symptom severity, previously unexplored, the SSC cohort displayed an expected decrease in the ratio among individuals with autism with increasing severity (19.3 for normative cases; 5.4 for severe cases; OR = 3.6; 95% CI = 1.58–10.11) (Fig. 4B; Additional file 7: Table S6). This trend was consistent in cases either with or without ID. Although we were not able to identify a decrease in the sex ratio depending on increasing total symptom severity in a consistent manner in the Korean cohort, we observed a decreasing trend in that ratio for individuals with autism and ID (6.2 for normative cases; 3.3 for severe cases; OR = 1.91; 95% CI = 0.88–4.63) (Fig. 4B). Additionally, the male-to-female ratio was the lowest for cases with ID and high total symptom severity in both the Korean and SSC cohorts (3.3 in the Korean, and 4.0 in the SSC cohort). Taken together, these findings suggest that males and females have different liability thresholds, therefore resulting in different sex ratios depending on ID and total symptom severity.

### Tolerance of polygenic burden for multiple autism traits in females within families

**Fig. 5:**
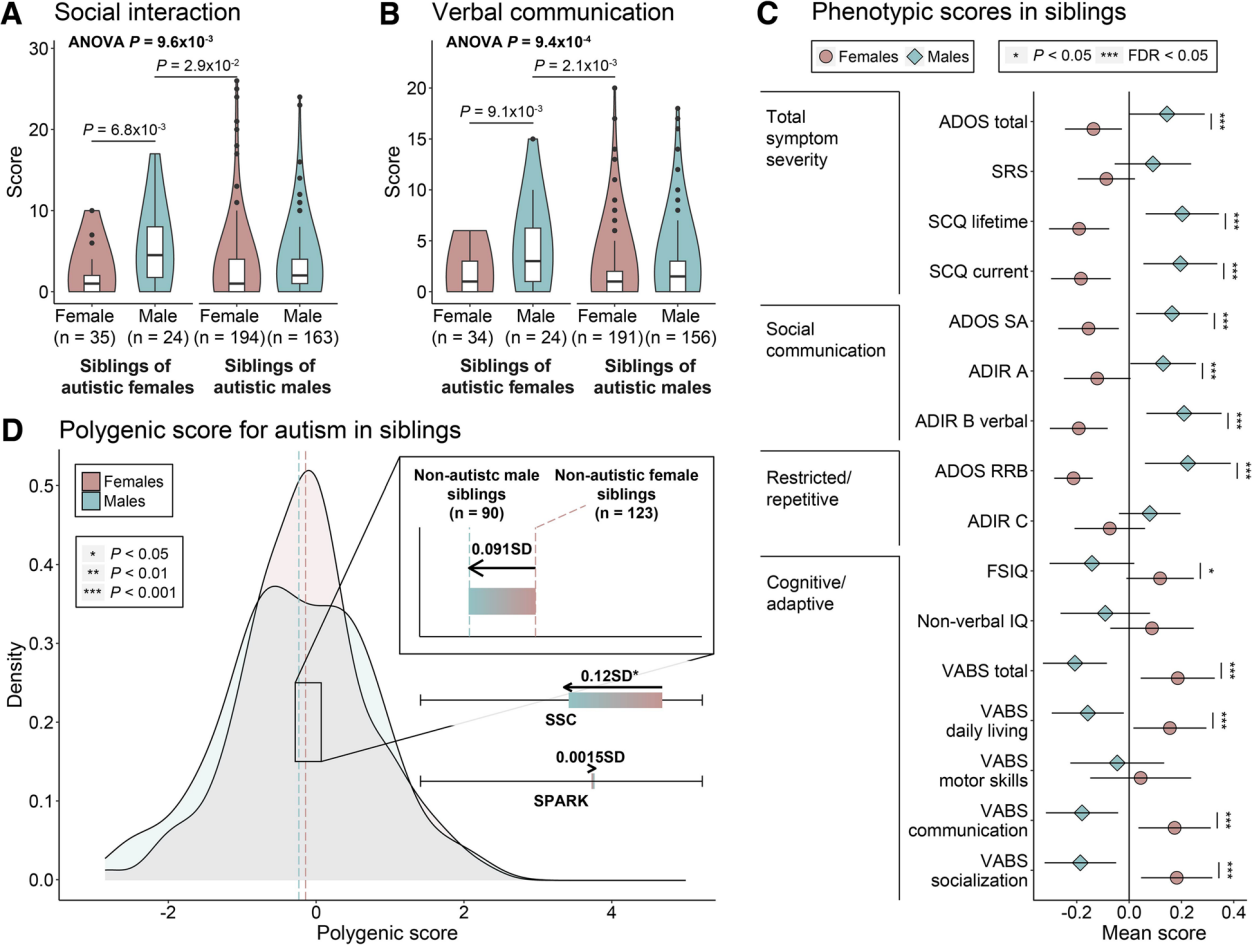
Effects of polygenic burden for various phenotypes in female and male siblings. **A**,**B** Comparison of social communication deficit scores across four groups of sibling-case sex pairs in siblings. Phenotypic scores include **A** abnormalities in reciprocal social interaction (ADIR A); **B** abnormalities in verbal communication (ADIR B verbal). Two-way ANOVA test, followed by Tukey’s multiple comparisons, was conducted and only adjusted *P* -values < 0.05 are displayed. Sex is represented by colors (dark pink, female siblings; blue-green, male siblings). **C** Comparison of z -transformed phenotypic scores including total symptom severity, social communication, restricted/repetitive behaviors, and cognitive/adaptive scores across sex in siblings. Two-sample t tests were used for the comparisons. Points represent mean scores and error bars represent the 95% CIs. Sex is represented by colors and shapes (blue-green circle, female siblings; dark pink diamond, male siblings) and the significance level is denoted by asterisk (“***”, FDR < 0.05; “*”, *P*  < 0.05).  **D** The distribution of PS in female and male siblings. The dashed lines and colored bar in the zoomed area correspond to the mean PS of each group in Korean families, while colored bars under the zoom-in box correspond to the group differences regarding PS in the SSC and SPARK cohorts. The direction of arrows represents the direction of enrichment from females to males. Group differences are standardized using the distribution of PS in families, and *P*- values were computed by two-sample t tests. Significance level is denoted by asterisk (“***”, *P*  < 0.001; “**”, *P*  < 0.01; “*”, *P*  < 0.05). Groups and sexes are represented by colors (dark pink, female siblings; blue-green, male siblings)

To elucidate sex-differential liability further, we compared phenotypic scores and polygenic burden across sexes within families. We compared the phenotypic scores in siblings, dividing them into four groups based on the sex of sibling-case pairs: female siblings of female cases (F_S_-F_C_), male siblings of female cases (M_S_-F_C_), female siblings of male cases (F_S_-M_C_), and male siblings of male cases (M_S_-M_C_). For a sex-differential liability threshold to exist, there would be a higher inherited genetic burden in families with female cases than those with male cases. Consequently, male siblings, who are less tolerant to this shared burden, would exhibit more severe clinical outcomes [64]. As expected, siblings in the M_S_-F_C_ pairs showed the most severe phenotypic scores, particularly for ADIR social interaction domain (ADIR A, *P* = 9.6 × 10^−3^, two-way ANOVA), and verbal sub-score in communication domain (ADIR B verbal, *P* = 9.4 × 10^−4^, two-way ANOVA), implicating lower social communication ability (Fig. 5A, B; Additional file 8: Table S7). This pattern was also observed in the replication cohort (Additional file 1: Fig. S6A-C; Additional file 8: Table S7). While these findings align with previous research [64], our analysis notably extends the scope by testing multiple subdomains of core features and development-associated features.

Next, we compared the clinical phenotypes between female and male siblings. Male siblings had significantly higher scores for seven out of nine clinical phenotypes related to autism core symptoms (total symptom severity, social communication and restricted interest and repetitive behaviors) and significantly lower scores for five out of seven cognitive/adaptive behaviors than female siblings (Fig. 5C; Additional file 8: Table S7), although sub-diagnostic. We observed the same pattern in the replication cohort (Additional file 1: Fig. S6C; Additional file 8: Table S7). These findings indicate that male siblings tend to exhibit more prominent autistic symptoms and encounter greater difficulties in cognitive and adaptive domains than their female counterparts.

Despite showing less severe scores on clinical phenotypes, female siblings displayed higher polygenic burden compared to that of male siblings, consistent with a previous finding [11] (0.091 SD, *P* = 0.49) (Fig. 5D; Additional file 8: Table S7). Regarding the SSC data, a significantly higher PS was observed for female siblings compared to that of male siblings (0.12 SD, *P* = 1.9 × 10^−2^).

**Fig. 6:**
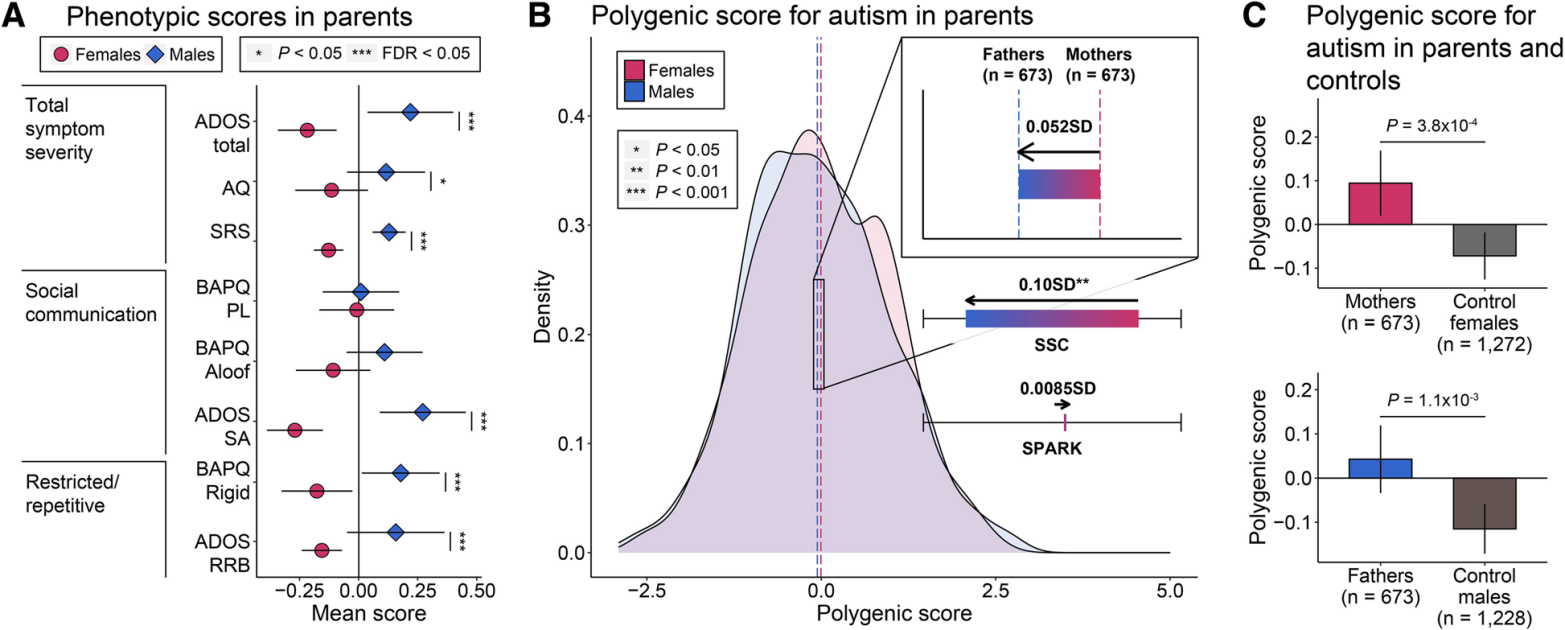
Effects of polygenic burden for various phenotypes in mothers and fathers.  **A** Comparison of z -transformed phenotypic scores, including total symptom severity, social communication, and restricted/repetitive behaviors. Two-sample t tests were conducted for the comparisons between groups. Points represent mean scores and error bars represent the 95% CIs. Group and sex are represented by colors and shapes (red circle, mothers; blue diamond, fathers), and the significance level is denoted by asterisk (“**”, FDR < 0.05; “*”, *P*  < 0.05). **B** The distribution of PS in parents. Dashed lines and colored bar in zoomed area correspond to the mean PS of each group in Korean families, while the colored bars under the zoom-in box correspond to the group differences regarding PS in the SSC and SPARK cohorts. The direction of arrows represents the direction of enrichment from females to males. Group differences are standardized using the distribution of PS in families and *P*-values were computed by two-sample t tests. The significance level is denoted by asterisk (“***”, *P*  < 0.001; “**”, *P*  < 0.01; “*”, *P*  < 0.05). The sex of parents is represented by different colors (red, mothers; blue, fathers).  **C** Comparison of PS between parents and control individuals from the general population. Two-sample t tests were conducted for group comparisons. Error bars represent the 95% CIs. Group and sex are represented by colors (red, mothers; blue, fathers; light gray, control female population; dark gray, control male population)

We next compared the clinical phenotypes between mothers and fathers. The fathers presented with higher scores for six out of eight core symptoms compared with mothers, except for the mean scores of BAPQ pragmatic language (PL) and BAPQ aloof (Fig. 6A; Additional file 9: Table S8). This observation is similar to the results for the SSC data (Additional file 1: Fig. S6D; Additional File 9: Table S8). Higher core symptom in fathers than mothers was also observed in the past study conducted with the NHS II cohort but limited to SRS only [61].

Both parents of children with autism carried an elevated PS burden relative to the general population (0.16 SD, *P* = 1.6 × 10^−6^) (Fig. 2D). However, the mothers had a higher polygenic burden than that of fathers (0.05 SD, *P* = 0.34) (Fig. 5B; Additional file 8: Table S7), consistent with a previous finding [11]. A higher PS in mothers than in fathers was also observed in the SSC cohort, and the difference was more pronounced relative to our own data (0.10 SD, *P* = 2.7 × 10^−3^). Both parents carried an elevated PS compared to control female (*P* = 3.8 × 10^−4^) and control male individuals (*P* = 1.1 × 10^−3^) of the general population (Fig. 6C; Additional file 9: Table S8); however, the difference was larger among mothers. Collectively, our findings first demonstrate that while females carried a higher polygenic burden than males, they exhibited relatively milder symptoms in families with autistic individuals. These results support the higher liability threshold in females, which makes females more tolerant to polygenic burden for autism core features and development-associated features.

## Discussion

Large-scale genetic studies of sex differences in autism have primarily been based on individuals of European ancestry. This study employed the WGS dataset and deep phenotyping collection of autism families of East Asian ancestry, broadening both the ancestral and the phenotypic diversity available for autism genetics studies. Our study covers the largest autism gene-phenotype data and the first investigation of sex differences of genetic factors and phenotype patterns in East Asian ancestry.

We provided evidence supporting a higher liability threshold in females, with a higher rate of de novo PTVs in females with autism than males with autism. We also observed female family members of autism cases exhibit less severe core symptoms and less impaired cognitive/adaptive ability even with a higher polygenic burden than males within families (Additional file 1: Fig. S7). In individuals with autism, we found that male-to-female ratio decreases when co-occurrence of ID or total symptom severity increases. Per cohort studied, polygenic burden in autistic individuals was enriched towards a sex of which has higher proportion of higher total symptom severity but lower chance of co-occurring ID. These findings corroborate the importance of taking into account not only comorbid ID but also total symptom severity of autism core symptom when examining sex differences in autism (Additional file 1: Fig. S7).

The female enrichment of de novo PTVs in individuals with autism was consistently observed across different cohorts. We found that the degree of female enrichment of de novo PTVs correlates with the presence of co-occurring ID. Notably, SSC exhibited the highest female bias in co-occurring ID (Fig. 3B) and, correspondingly, the most robust female enrichment of de novo PTVs (Fig. 2C). This trend was similarly observed in the Korean cohort, although it did not reach the statistical significance. However, the power analysis estimates that a threefold increase in the number of female samples would achieve 80% statistical power for this difference (Additional file 1: Fig. S3).

In contrast, the SPARK cohort, which showed the least female bias in co-occurring ID, did not show significant female enrichment of de novo PTVs even with an increased sample size up to 5000 female cases (Additional file 1: Fig. S3). This disparity across cohorts can be in part be attributed to a greater rate of diagnoses among females with ID than without ID [2, 65], which may result from current diagnostic biases in the content or implementation of clinical diagnostic instruments. These results reflect the influence of ascertainment biases and females’ masking behaviors, as evidenced by varying male-to-female ratios in autistic children (5.6 in Korean, 6.2 in SSC, and 3.8 in SPARK) (Fig. 1C). However, a weak female bias in de novo PTVs persisted even in cases without ID (Additional file 1: Fig. S8).

We showed that European-derived polygenic score successfully separates autistic individuals from non-autistic individuals of East Asian ancestry (Fig. 2D, E). Though we confirmed consistent patterns of PS across different methodologies (Additional file 1: Fig. S2), generalizability of European-derived PS still warrants further evaluation in diverse cohorts and ancestries. Sex differences in PS varied across cohorts. We found that inconsistency across cohorts stemmed from varying proportions of severe core symptoms and non-ID conditions between sexes. These findings highlight the importance of accounting for phenotypic heterogeneity when evaluating sex differences in genetic predispositions. After correcting for FSIQ and SRS, no sex differences in PS were found, which contradicts the expectations from sex-differential liability model. This result supports the possibility of other liability model which hypothesizes that sex differences can be attributed to not only a higher threshold in females but also a greater genetic variability in males [63, 66, 67]. For more definite conclusions, the increased sample size of female cases is needed, especially when stratifying by phenotypes. Additionally, there lies a possibility that the GWAS datasets utilized for PS calculation, predominantly comprising males, might result in an underestimation of PS in autistic females.

In a subset of autism cases with a more severe clinical presentation, comorbid ID or high total symptom severity, the male-to-female sex ratio decreased. This result aligns with the principal assumption of a sex-differential liability threshold in autism [63]. We selected two fundamental clinical features that have known associations with genetic variants [6, 9, 10, 12, 59]; ID and total symptom severity, measured by SRS. However, deconvolution of other clinical measures or various comorbidities would give us an invaluable perspective on the relationship between neurodiversity and genetic liability. Though SRS was not assessable in the SPARK dataset, we assume that individuals with autism in that cohort would have a rather distinct clinical landscape given the smaller male-to-female autism ratio. Considering the large number of female probands that comprise the SPARK cohort, exploring sex differences in SPARK would be important for better understanding the sex-differential liability in autism.

Among unaffected family members, female siblings and mothers harbor elevated polygenic burden but exhibit less severe symptoms compared to male siblings and fathers. These results show that females have a higher liability threshold for autism than males, implying a female-specific biological mechanism that buffers polygenic burden, which was not observed in autism cases. Considering that siblings and parents are less likely to carry autism-associated de novo variants, investigation of their sex differences may have enabled the approximate comparison across sexes with the same variance [63, 66, 67], hinting the underlying sex-differential liability model.

Our analysis provides evidence to support the differing liability threshold for autism across sexes. We also found that the varying phenotype severity in female and male individuals with autism is a latent factor that shapes the associated sex differences regarding genetic burden. Future GWAS studies with a more balanced representation of autistic females and diverse ancestries and large-scale investigation on sex differences, along with the collection of data from clinically diagnosed and sub-diagnostic females are warranted to better understand female-specific autism biology. We believe that addressing these questions will provide vital insights into the neurobiological mechanisms contributing to sex bias in autism.

## Conclusions

Our work supports a complex model of sex-differential liability in autism, where combinatorial effects of comorbid ID and total symptom severity affect sex differences of genetic burden in autistic individuals. Within family, we observed higher tolerance of inherited risk in females for multiple clinical features than males, implying the higher liability threshold in females. These results first exemplify and emphasize the importance of taking phenotypic heterogeneity and family-based study into account to understand sex differences in autism.

## Supplementary Information

Additional file 1: Fig. S1 Correlation between *de novo* burden and paternal age and *de novo* MIS burden test. A, Correlation of paternal age with the number of *de novo* variants. The number of DNVs was adjusted with paternal age of birth for comparison of DNV burden across groups and sexes. R^2^ and *P*-values were computed from a linear regression. B-C, Comparison of the *de novo *MIS in MPC ≥ 2 genes adjusted for paternal age at birth across Korean, SSC, and SPARK cohorts; B, between individuals with autism and non-autistic siblings; C, between sex in individuals with autism. The y axis indicates the average number of variants. *P*-values were computed by one-sided exact binomial test. Groups and sexes are represented by colors. Fig. S2| PS from different methodologies. A, Correlation of PS across 4 different methodologies for calculating PS. R^2^ and *P*-values were computed from a linear regression. Autism status is represented by colorsand the significance of *P* < 1.0x10^-12^ is denoted by ‘***’. Fig. S3| Power calculation of *de novo* burden test. A-B, Power estimation for risk ratioin Korean, SSC, and SPARK cohorts; A, for *de novo* PTVs and MIS across individuals with autism and non-autistic siblings; B, for *de novo* PTVs in individuals across sex. The power of RR was computed by binom.powerfunction in R. The success probabilities under the null hypothesis are the ratio of individuals with autism out of total samples. The success probabilities under the alternative hypothesis are the ratio of DNVs. The number of independent trials is the sample size. Power estimation was iterated followed by the increase in sample size. X axis of the figure was calculated by multiplying the ratio of individuals with autism to the sample size. Red vertical lines display the total number of cases in the current datasets. Type of variant is represented by colors. Fig. S4| Sex-specific autism-associated genes. A, TADA workflow for identification of sex-specific autism-associated genes. B, Biological pathways enriched for TADA female genes, male genes, and both genes. Each row represents a different biological pathway, and the size of the circle in each column corresponds to the gene ratio involved in each pathway, colored by the adjusted p-value significance. C, The network of enriched biological pathways of female-only genes, male-only genes and both genes. The number of genes involved is represented by size of circle and whether the pathway is enriched by female-only genes, male-only genes or both genes is represented by colors. Fig. S5| Sex-differential liability threshold model. A, Sex-differential liability threshold for autism. Sexes are represented by colors. B, Relationship between sex-differential liability threshold and male-to-female sex ratio. Fig. S6| Sex differences of phenotypic scores in siblings and parents in the replication cohort. A-B, Comparison of total symptom severity and developmental age across 4 groups of sibling-case sex pairs in siblings in SPARK. Phenotypic scores include A, SCQ lifetime; B, age of first word. Two-way ANOVA test, followed by Tukey’s multiple comparisons, was conducted and only adjusted *P*-values < 0.05 are displayed. Sex is represented by colors. C, Comparison of z-transformed phenotypic scores including total symptom severity, social communication, restricted/repetitive behaviors, and cognitive/adaptive scores across sex in siblings in SSC, and SPARK. Two-sample t tests were used for contrasts. Points represent mean scores, and error bars represent the 95% CIs. Sex is represented by colors and shapesand the significance level is denoted by asterisk. D, Comparison of z-transformed total symptom severity scores across sex in parents in SSC. Two-sample t tests were used for contrasts. Points represent mean scores, and error bars represent the 95% CIs. Sex is represented by colors and shapesand the significance level is denoted by asterisk. Fig. S7| Key findings under the sex-differential liability threshold model. A, Key findings in this study under the sex-differential liability threshold model. Sexes are represented by colors. Fig. S8| Effects of ID and total symptom severity on sex differences in *de novo* and polygenic burden. A-B, Comparison of the *de novo* PTVs in constrained genes, adjusted for paternal age at birth across Korean autism, SSC, and SPARK cohorts between sex; A, among children with autism and ID; B, among children with autism and without ID. The y axis indicates the average number of variants. The *P*-values were computed by one-sided exact binomial test. Groups and sexes are represented by colors.

Additional file 2: Table S1. This table contains data overview including the number of samples (Table S1A), phenotype coverage (Table S1B), and phenotype and genetic burden per sample (Table S1C-E) in Korean, SSC, and SPARK cohorts

Additional file 3: Table S2. This table lists de novo variants identified in the Korean cohort (Table S2A) and contains the comparison results of genetic burden across groups in Korean, SSC, and SPARK cohorts: comparison of de novo burden across groups (Table S2B), power estimation for comparison of de novo burden across groups (Table S2C-E), comparison of polygenic burden between Korean autism and KoGES (Table S2F), and comparison of polygenic burden across groups (Table S2G)

Additional file 4: Table S3. This table contains the results of sex differences in autistic individuals in Korean, SSC, and SPARK cohorts: comparison of de novo burden across sexes (Table S3A), power estimation for comparison of de novo burden across sexes (Table S3B-D), comparison of polygenic burden across sexes (Table S3E), and comparison of phenotypic scores across sexes (Table S3F)

Additional file 5: Table S4. This table contains the results of TADA gene discovery in females and males and downstream GO functional analysis: the number of autistic individuals used in TADA analysis (Table S4A), results of TADA in females (Table S4B) and males (Table S4C), and GO results for identified genes (Table S4D)

Additional file 6: Table S5. This table contains the results of gene-phenotype associations in Korean, SSC, and SPARK cohorts: comparison of de novo burden across groups depending on comorbid ID (Table S5A), relative enrichment of autistic females than autistic males with comorbid ID (Table S5B) or with high SRS severity (Table S5C), comparison of polygenic burden across groups depending on comorbid ID and SRS severity (Table S5D), and correlation results of polygenic burden across sexes correcting for IQ and SRS (Table S5E)

Additional file 7: Table S6. This table lists male-to-female sex ratios in autistic individuals across ID co-occurrence (Table S6A) and SRS severity (Table S6B) in Korean, SSC, and SPARK cohorts

Additional file 8: Table S7. This table contains the results of sex differences in unaffected siblings in Korean, SSC, and SPARK cohorts: comparison of phenotypic scores across sibling-case sex pairs (Table S7A), and across sexes of themselves (Table S7B), and comparison of polygenic burden across sexes (Table S7C)

Additional file 9: Table S8. This table contains the results of sex differences parents in Korean, and SSC cohorts: comparison of phenotypic scores across sexes (Table S8A), and comparison of polygenic burden across sexes (Table S8B)

## Data Availability

We share the list of de novo variants from Korean autism cases used in this study in Additional file 3: Table S2A. The raw genomic data (fastq, VCF) of the Korean ASD WGS families are available for data sharing by application from qualified researchers, in accordance with participant consent and privacy protections. Researchers interested in accessing the Korean WGS datasets may submit requests to the corresponding author, Dr. Heejeong Yoo. To ensure responsible use of the data, we ask requesters to provide a detailed research plan outlining the proposed analyses and data anonymization procedures. This plan will undergo review by both the IRB and the data sharing committee at Seoul National University Bundang Hospital. Following approval, the requester will be added to our IRB as a collaborator, facilitating secure data sharing. The review process typically concludes within two months of submission. We are committed to sharing data securely and efficiently with approved researchers. Extended data generated in this study are available in the supplementary materials accompanying this manuscript. The KoGES WGS and phenotypic data can be obtained by applying through the National Project of Bio Big Data (www.nih.go.kr/biobank/, accession ID: CODA D22001). Genetic and phenotypic data for the SSC and SPARK cohorts used in this study are accessible by applying at https://base.sfari.org. GWAS summary statistics are available from the Psychiatric Genomics Consortium (PGC) (https://pgc.unc.edu/for-researchers/download-results/) (PMID: 30,804,558). The code for major analyses and for generating Figs. 2, 3, 4, 5, and 6 is available at Zenodo (https://doi.org/10.5281/zenodo.11178096) [68].

## Abbreviations

WGS: Whole-genome sequencing
PTV: Protein-truncating variant
MIS: Missense variant
DNV: De novo variant
PS: Polygenic score
TADA: Transmitted and de novo association gene discovery
GWAS: Genome Wide Association Study
ID: Intellectual disability
SRS: Social responsiveness scale
VABS: Vineland adaptive behavior scale

## References

[1] Fombonne E. Epidemiology of pervasive developmental disorders. Pediatr Res. 2009;65(6):591–8. 19218885 10.1203/PDR.0b013e31819e7203

[2] Loomes R, Hull L, Mandy WPL. What is the male-to-female ratio in autism spectrum disorder? A Systematic Review and Meta-Analysis. J Am Acad Child Adolesc Psychiatry. 2017;56(6):466–74. 28545751 10.1016/j.jaac.2017.03.013

[3] Maenner MJ, Shaw KA, Bakian AV, Bilder DA, Durkin MS, Esler A, et al. Prevalence and characteristics of autism spectrum disorder among children aged 8 years - autism and developmental disabilities monitoring network, 11 sites, United States, 2018. MMWR Surveill Summ. 2021;70(11):1–16. 34855725 10.15585/mmwr.ss7011a1 PMC8639024

[4] Werling DM. The role of sex-differential biology in risk for autism spectrum disorder. Biol Sex Differ. 2016;7:58. 27891212 10.1186/s13293-016-0112-8 PMC5112643

[5] Jacquemont S, Coe BP, Hersch M, Duyzend MH, Krumm N, Bergmann S, et al. A higher mutational burden in females supports a “female protective model” in neurodevelopmental disorders. Am J Hum Genet. 2014;94(3):415–25. 24581740 10.1016/j.ajhg.2014.02.001 PMC3951938

[6] Satterstrom FK, Kosmicki JA, Wang J, Breen MS, De Rubeis S, An J-Y, et al. Large-scale exome sequencing study implicates both developmental and functional changes in the neurobiology of autism. Cell. 2020;180(3):568–84 e23. 31981491 10.1016/j.cell.2019.12.036 PMC7250485

[7] Fu JM, Satterstrom FK, Peng M, Brand H, Collins RL, Dong S, et al. Rare coding variation provides insight into the genetic architecture and phenotypic context of autism. Nat Genet. 2022;54(9):1320–31. 35982160 10.1038/s41588-022-01104-0 PMC9653013

[8] Polyak A, Rosenfeld JA, Girirajan S. An assessment of sex bias in neurodevelopmental disorders. Genome Med. 2015;7(1):94. 26307204 10.1186/s13073-015-0216-5 PMC4549901

[9] Antaki D, Guevara J, Maihofer AX, Klein M, Gujral M, Grove J, et al. A phenotypic spectrum of autism is attributable to the combined effects of rare variants, polygenic risk and sex. Nat Genet. 2022;54(9):1284–92. 35654974 10.1038/s41588-022-01064-5 PMC9474668

[10] Buja A, Volfovsky N, Krieger AM, Lord C, Lash AE, Wigler M, et al. Damaging de novo mutations diminish motor skills in children on the autism spectrum. Proc Natl Acad Sci U S A. 2018;115(8):E1859–66. 29434036 10.1073/pnas.1715427115 PMC5828599

[11] Wigdor EM, Weiner DJ, Grove J, Fu JM, Thompson WK, Carey CE, et al. The female protective effect against autism spectrum disorder. Cell Genom. 2022;2(6): 100134. 36778135 10.1016/j.xgen.2022.100134 PMC9903803

[12] Warrier V, Zhang X, Reed P, Havdahl A, Moore TM, Cliquet F, et al. Genetic correlates of phenotypic heterogeneity in autism. Nat Genet. 2022;54(9):1293–304. 35654973 10.1038/s41588-022-01072-5 PMC9470531

[13] Trost B, Thiruvahindrapuram B, Chan AJS, Engchuan W, Higginbotham EJ, Howe JL, et al. Genomic architecture of autism from comprehensive whole-genome sequence annotation. Cell. 2022;185(23):4409-27.e18. 36368308 10.1016/j.cell.2022.10.009 PMC10726699

[14] Fischbach GD, Lord C. The Simons Simplex Collection: a resource for identification of autism genetic risk factors. Neuron. 2010;68(2):192–5. 20955926 10.1016/j.neuron.2010.10.006

[15] SPARK: A US Cohort of 50,000 Families to Accelerate Autism Research. Neuron. 2018;97(3):488–93. 10.1016/j.neuron.2018.01.015 PMC7444276 29420931

[16] Ratto AB, Kenworthy L, Yerys BE, Bascom J, Wieckowski AT, White SW, et al. What about the girls? Sex-Based Differences in Autistic Traits and Adaptive Skills. J Autism Dev Disord. 2018;48(5):1698–711. 29204929 10.1007/s10803-017-3413-9 PMC5925757

[17] Rea HM, Øien RA, Shic F, Webb SJ, Ratto AB. Sex differences on the ADOS-2. J Autism Dev Disord. 2023;53(7):2878–90. 35451672 10.1007/s10803-022-05566-3 PMC10074828

[18] Lai MC, Szatmari P. Sex and gender impacts on the behavioural presentation and recognition of autism. Curr Opin Psychiatry. 2020;33(2):117–23. 31815760 10.1097/YCO.0000000000000575

[19] Rolland T, Cliquet F, Anney RJL, Moreau C, Traut N, Mathieu A, et al. Phenotypic effects of genetic variants associated with autism. Nat Med. 2023;29(7):1671–80. 37365347 10.1038/s41591-023-02408-2 PMC10353945

[20] Kim JH, Koh IG, Lee H, Lee GH, Song DY, Kim SW, et al. Short tandem repeat expansions in cortical layer-specific genes implicate in phenotypic severity and adaptability of autism spectrum disorder. Psychiatry Clin Neurosci. 2024;78(7):405–15. 38751214 10.1111/pcn.13676 PMC11488627

[21] Kim Y, Han BG. Cohort profile: the Korean Genome and Epidemiology Study (KoGES) consortium. Int J Epidemiol. 2017;46(2): e20. 27085081 10.1093/ije/dyv316 PMC5837648

[22] Miller NA, Farrow EG, Gibson M, Willig LK, Twist G, Yoo B, et al. A 26-hour system of highly sensitive whole genome sequencing for emergency management of genetic diseases. Genome Med. 2015;7:100. 26419432 10.1186/s13073-015-0221-8 PMC4588251

[23] Pedersen BS, Quinlan AR. Who’s who? Detecting and resolving sample anomalies in human DNA sequencing studies with Peddy. Am J Hum Genet. 2017;100(3):406–13. 28190455 10.1016/j.ajhg.2017.01.017 PMC5339084

[24] Werling DM, Brand H, An JY, Stone MR, Zhu L, Glessner JT, et al. An analytical framework for whole-genome sequence association studies and its implications for autism spectrum disorder. Nat Genet. 2018;50(5):727–36. 29700473 10.1038/s41588-018-0107-y PMC5961723

[25] Samocha KE, Robinson EB, Sanders SJ, Stevens C, Sabo A, McGrath LM, et al. A framework for the interpretation of de novo mutation in human disease. Nat Genet. 2014;46(9):944–50. 25086666 10.1038/ng.3050 PMC4222185

[26] McGrath JJ, Petersen L, Agerbo E, Mors O, Mortensen PB, Pedersen CB. A comprehensive assessment of parental age and psychiatric disorders. JAMA Psychiat. 2014;71(3):301–9. 10.1001/jamapsychiatry.2013.4081 24452535

[27] Reichenberg A, Gross R, Weiser M, Bresnahan M, Silverman J, Harlap S, et al. Advancing paternal age and autism. Arch Gen Psychiatry. 2006;63(9):1026–32. 16953005 10.1001/archpsyc.63.9.1026

[28] Grether JK, Anderson MC, Croen LA, Smith D, Windham GC. Risk of autism and increasing maternal and paternal age in a large north American population. Am J Epidemiol. 2009;170(9):1118–26. 19783586 10.1093/aje/kwp247

[29] Hultman CM, Sandin S, Levine SZ, Lichtenstein P, Reichenberg A. Advancing paternal age and risk of autism: new evidence from a population-based study and a meta-analysis of epidemiological studies. Mol Psychiatry. 2011;16(12):1203–12. 21116277 10.1038/mp.2010.121

[30] Karczewski KJ, Francioli LC, Tiao G, Cummings BB, Alföldi J, Wang Q, et al. The mutational constraint spectrum quantified from variation in 141,456 humans. Nature. 2020;581(7809):434–43. 32461654 10.1038/s41586-020-2308-7 PMC7334197

[31] Grove J, Ripke S, Als TD, Mattheisen M, Walters RK, Won H, et al. Identification of common genetic risk variants for autism spectrum disorder. Nat Genet. 2019;51(3):431–44. 30804558 10.1038/s41588-019-0344-8 PMC6454898

[32] Ge T, Chen CY, Ni Y, Feng YA, Smoller JW. Polygenic prediction via Bayesian regression and continuous shrinkage priors. Nat Commun. 2019;10(1):1776. 30992449 10.1038/s41467-019-09718-5 PMC6467998

[33] Lloyd-Jones LR, Zeng J, Sidorenko J, Yengo L, Moser G, Kemper KE, et al. Improved polygenic prediction by Bayesian multiple regression on summary statistics. Nat Commun. 2019;10(1):5086. 31704910 10.1038/s41467-019-12653-0 PMC6841727

[34] Privé F, Arbel J, Vilhjálmsson BJ. LDpred2: better, faster, stronger. Bioinformatics. 2020;36(22–23):5424–31. 10.1093/bioinformatics/btaa1029 PMC8016455 33326037

[35] Choi SW, O’Reilly PF. PRSice-2: polygenic risk score software for biobank-scale data. GigaScience. 2019;8(7):giz082. 31307061 10.1093/gigascience/giz082 PMC6629542

[36] Pain O, Glanville KP, Hagenaars SP, Selzam S, Fürtjes AE, Gaspar HA, et al. Evaluation of polygenic prediction methodology within a reference-standardized framework. PLoS Genet. 2021;17(5): e1009021. 33945532 10.1371/journal.pgen.1009021 PMC8121285

[37] He X, Sanders SJ, Liu L, De Rubeis S, Lim ET, Sutcliffe JS, et al. Integrated model of de novo and inherited genetic variants yields greater power to identify risk genes. PLoS Genet. 2013;9(8):e1003671. 23966865 10.1371/journal.pgen.1003671 PMC3744441

[38] Lord C, Rutter M, DiLavore P, Risi S, Gotham K, Bishop S. Autism diagnostic observation schedule–(ADOS-2), 2nd Edn. Los Angeles: Western Psychological Services; 2012.

[39] Yoo H, Bong G, Kwak Y, Lee M, Cho S, Kim B, et al. Korean autism diagnostic observation schedule-2 (K-ADOS-2). Seoul: Hakjisa; 2018.

[40] Constantino JN, Gruber CP. Social responsiveness scale: SRS-2. Los Angeles: Western Psychological Services; 2012.

[41] Rutter M, Bailey A, Lord C. The social communication questionnaire. Los Angeles: Western Psychological Services; 2003.

[42] Kim J-H, Sunwoo H-J, Park S-B, Noh D-H, Jung YK, Cho I-H, et al. A validation study of the Korean version of social communication questionnaire. Journal of the Korean Academy of Child and Adolescent Psychiatry. 2015;26(3):197–208.

[43] Baron-Cohen S, Wheelwright S, Skinner R, Martin J, Clubley E. The autism-spectrum quotient (AQ): Evidence from asperger syndrome/high-functioning autism, malesand females, scientists and mathematicians. J Autism Dev Disord. 2001;31:5–17. 11439754 10.1023/a:1005653411471

[44] Hurley RS, Losh M, Parlier M, Reznick JS, Piven J. The broad autism phenotype questionnaire. J Autism Dev Disord. 2007;37:1679–90. 17146701 10.1007/s10803-006-0299-3

[45] Wechsler D. WPPSI-III administration and scoring manual: psychological corporation. 2002.

[46] Wechsler D. Wechsler intelligence scale for children–Fourth Edition (WISC-IV). San Antonio: Harcourt Assessment; 2003.

[47] Roid GH, Miller LJ. Leiter international performance scale-revised (Leiter-R). Wood Dale: Stoelting. 1997;10.

[48] Sparrow S, Cicchetti D, Balla D. Vineland Adaptive Behavior Scales–2nd edition (VABS-II). Livonia: Pearson Assessments; 2005.

[49] Samocha KE, Kosmicki JA, Karczewski KJ, O’Donnell-Luria AH, Pierce-Hoffman E, MacArthur DG, et al. Regional missense constraint improves variant deleteriousness prediction. bioRxiv. 2017. p. 148353. Available from: 10.1101/148353.

[50] Zhang Y, Li Y, Guo R, Xu W, Liu X, Zhao C, et al. Genetic diagnostic yields of 354 Chinese ASD children with rare mutations by a pipeline of genomic tests. Front Genet. 2023;14: 1108440. 37035742 10.3389/fgene.2023.1108440 PMC10076746

[51] Lai MC, Lombardo MV, Baron-Cohen S. Autism. Lancet. 2014;383(9920):896–910. 24074734 10.1016/S0140-6736(13)61539-1

[52] Geschwind DH. Advances in autism. Annu Rev Med. 2009;60:367–80. 19630577 10.1146/annurev.med.60.053107.121225 PMC3645857

[53] An JY, Lin K, Zhu L, Werling DM, Dong S, Brand H, et al. Genome-wide de novo risk score implicates promoter variation in autism spectrum disorder. Science. 2018;362(6420):eaat6576. 30545852 10.1126/science.aat6576 PMC6432922

[54] Werling DM, Pochareddy S, Choi J, An JY, Sheppard B, Peng M, et al. Whole-genome and RNA sequencing reveal variation and transcriptomic coordination in the developing human prefrontal cortex. Cell Rep. 2020;31(1): 107489. 32268104 10.1016/j.celrep.2020.03.053 PMC7295160

[55] Iossifov I, O’Roak BJ, Sanders SJ, Ronemus M, Krumm N, Levy D, et al. The contribution of de novo coding mutations to autism spectrum disorder. Nature. 2014;515(7526):216–21. 25363768 10.1038/nature13908 PMC4313871

[56] De Rubeis S, He X, Goldberg AP, Poultney CS, Samocha K, Cicek AE, et al. Synaptic, transcriptional and chromatin genes disrupted in autism. Nature. 2014;515(7526):209–15. 25363760 10.1038/nature13772 PMC4402723

[57] Weiner DJ, Wigdor EM, Ripke S, Walters RK, Kosmicki JA, Grove J, et al. Polygenic transmission disequilibrium confirms that common and rare variation act additively to create risk for autism spectrum disorders. Nat Genet. 2017;49(7):978–85. 28504703 10.1038/ng.3863 PMC5552240

[58] Sanders SJ, He X, Willsey AJ, Ercan-Sencicek AG, Samocha KE, Cicek AE, et al. Insights into autism spectrum disorder genomic architecture and biology from 71 risk loci. Neuron. 2015;87(6):1215–33. 26402605 10.1016/j.neuron.2015.09.016 PMC4624267

[59] Cirnigliaro M, Chang TS, Arteaga SA, Pérez-Cano L, Ruzzo EK, Gordon A, et al. The contributions of rare inherited and polygenic risk to ASD in multiplex families. Proc Natl Acad Sci U S A. 2023;120(31): e2215632120. 37506195 10.1073/pnas.2215632120 PMC10400943

[60] Constantino JN, Davis SA, Todd RD, Schindler MK, Gross MM, Brophy SL, et al. Validation of a brief quantitative measure of autistic traits: comparison of the social responsiveness scale with the autism diagnostic interview-revised. J Autism Dev Disord. 2003;33(4):427–33. 12959421 10.1023/a:1025014929212

[61] Lyall K, Constantino JN, Weisskopf MG, Roberts AL, Ascherio A, Santangelo SL. Parental social responsiveness and risk of autism spectrum disorder in offspring. JAMA Psychiat. 2014;71(8):936–42. 10.1001/jamapsychiatry.2014.476 PMC4126195 25100167

[62] Falconer DS. The inheritance of liability to certain diseases, estimated from the incidence among relatives. Ann Hum Genet. 1965;29(1):51–76.

[63] Dougherty JD, Marrus N, Maloney SE, Yip B, Sandin S, Turner TN, et al. Can the “female protective effect” liability threshold model explain sex differences in autism spectrum disorder? Neuron. 2022;110(20):3243–62. 35868305 10.1016/j.neuron.2022.06.020 PMC9588569

[64] Robinson EB, Lichtenstein P, Anckarsäter H, Happé F, Ronald A. Examining and interpreting the female protective effect against autistic behavior. Proc Natl Acad Sci U S A. 2013;110(13):5258–62. 23431162 10.1073/pnas.1211070110 PMC3612665

[65] Harrop C, Tomaszewski B, Putnam O, Klein C, Lamarche E, Klinger L. Are the diagnostic rates of autistic females increasing? An examination of state-wide trends. J Child Psychol Psychiatry. 2024;65(7):973–83. 10.1111/jcpp.13939 PMC11161335 38181181

[66] Sandin S, Yip BHK, Yin W, Weiss LA, Dougherty JD, Fass S, et al. Examining Sex Differences in Autism Heritability. JAMA Psychiatr. 2024;81(7):673–80. 10.1001/jamapsychiatry.2024.0525 PMC11024771 38630491

[67] Girirajan S, Rosenfeld JA, Coe BP, Parikh S, Friedman N, Goldstein A, et al. Phenotypic heterogeneity of genomic disorders and rare copy-number variants. N Engl J Med. 2012;367(14):1321–31. 22970919 10.1056/NEJMoa1200395 PMC3494411

[68] Soo-Whee K, Heyji L, Gang-Hee L. Whole-genome sequencing and phenotype data analysis, v1 ed. Zenodo. 2024. Available from: 10.5281/zenodo.11178096.

