# Supplementary material for: Whole genome sequencing analysis identifies sex differences of familial pattern contributing to phenotypic diversity in autism
Source: Genome Med. 2024 Sep 27;16:114. doi: 10.1186/s13073-024-01385-6 (PMC11429951; doi:10.1186/s13073-024-01385-6)
Supplement: Supplementary file 1 — Additional file 1: Fig. S1 Correlation between de novo burden and paternal age and de novo MIS burden test. A, Correlation of paternal age with the number of de novo variants. The number of DNVs was adjusted with paternal age of birth for comparison of DNV burden across groups and sexes. R2 and P-values were computed from a linear regression. B-C, Comparison of the de novo MIS in MPC ≥ 2 genes adjusted for paternal age at birth across Korean, SSC, and SPARK cohorts; B, between individuals with autism and non-autistic siblings; C, between sex in individuals with autism. The y axis indicates the average number of variants. P-values were computed by one-sided exact binomial test. Groups and sexes are represented by colors. Fig. S2| PS from different methodologies. A, Correlation of PS across 4 different methodologies for calculating PS. R2 and P-values were computed from a linear regression. Autism status is represented by colorsand the significance of P < 1.0x10^-12^ is denoted by ‘***’. Fig. S3| Power calculation of de novo burden test. A-B, Power estimation for risk ratioin Korean, SSC, and SPARK cohorts; A, for de novo PTVs and MIS across individuals with autism and non-autistic siblings; B, for de novo PTVs in individuals across sex. The power of RR was computed by binom.powerfunction in R. The success probabilities under the null hypothesis are the ratio of individuals with autism out of total samples. The success probabilities under the alternative hypothesis are the ratio of DNVs. The number of independent trials is the sample size. Power estimation was iterated followed by the increase in sample size. X axis of the figure was calculated by multiplying the ratio of individuals with autism to the sample size. Red vertical lines display the total number of cases in the current datasets. Type of variant is represented by colors. Fig. S4| Sex-specific autism-associated genes. A, TADA workflow for identification of sex-specific autism-associated genes. B, Biological p [file 13073_2024_1385_MOESM1_ESM.docx]

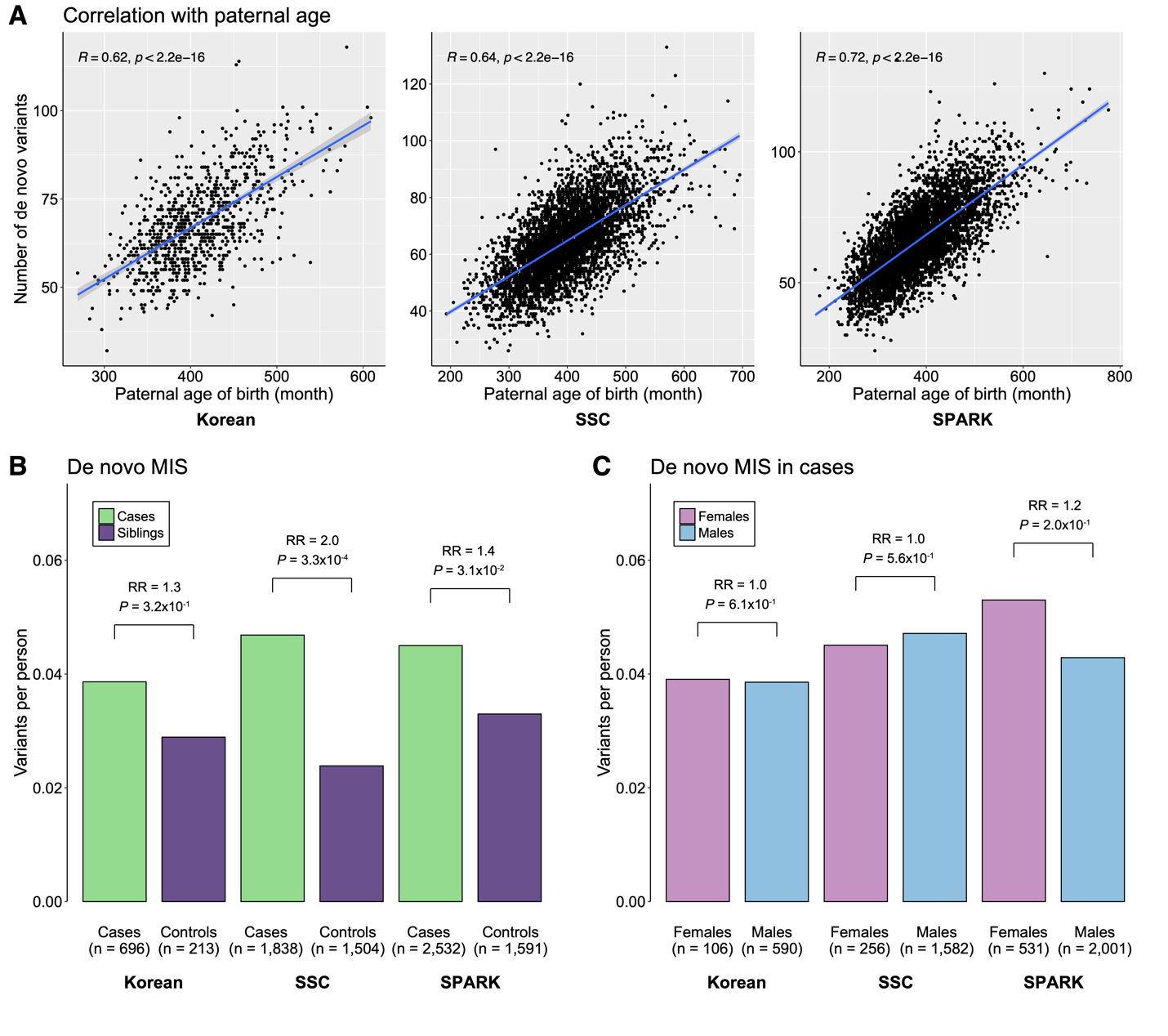


**Fig. S1|** **Correlation between *de novo* burden and paternal age and *de novo* MIS burden test**

**A,** Correlation of paternal age with the number of *de novo* variants. The number of DNVs was adjusted with paternal age of birth for comparison of DNV burden across groups and sexes. R^2^ and *P*-values were computed from a linear regression.

**B-C,** Comparison of the *de novo* MIS in MPC ≥ 2 genes adjusted for paternal age at birth across Korean, SSC, and SPARK cohorts; **B,** between individuals with autism and non-autistic siblings; **C,** between sex in individuals with autism. The y axis indicates the average number of variants. *P*-values were computed by one-sided exact binomial test. Groups and sexes are represented by colors (green, autism cases; purple, non-autistic siblings; pink, female cases; light blue, male cases).

**
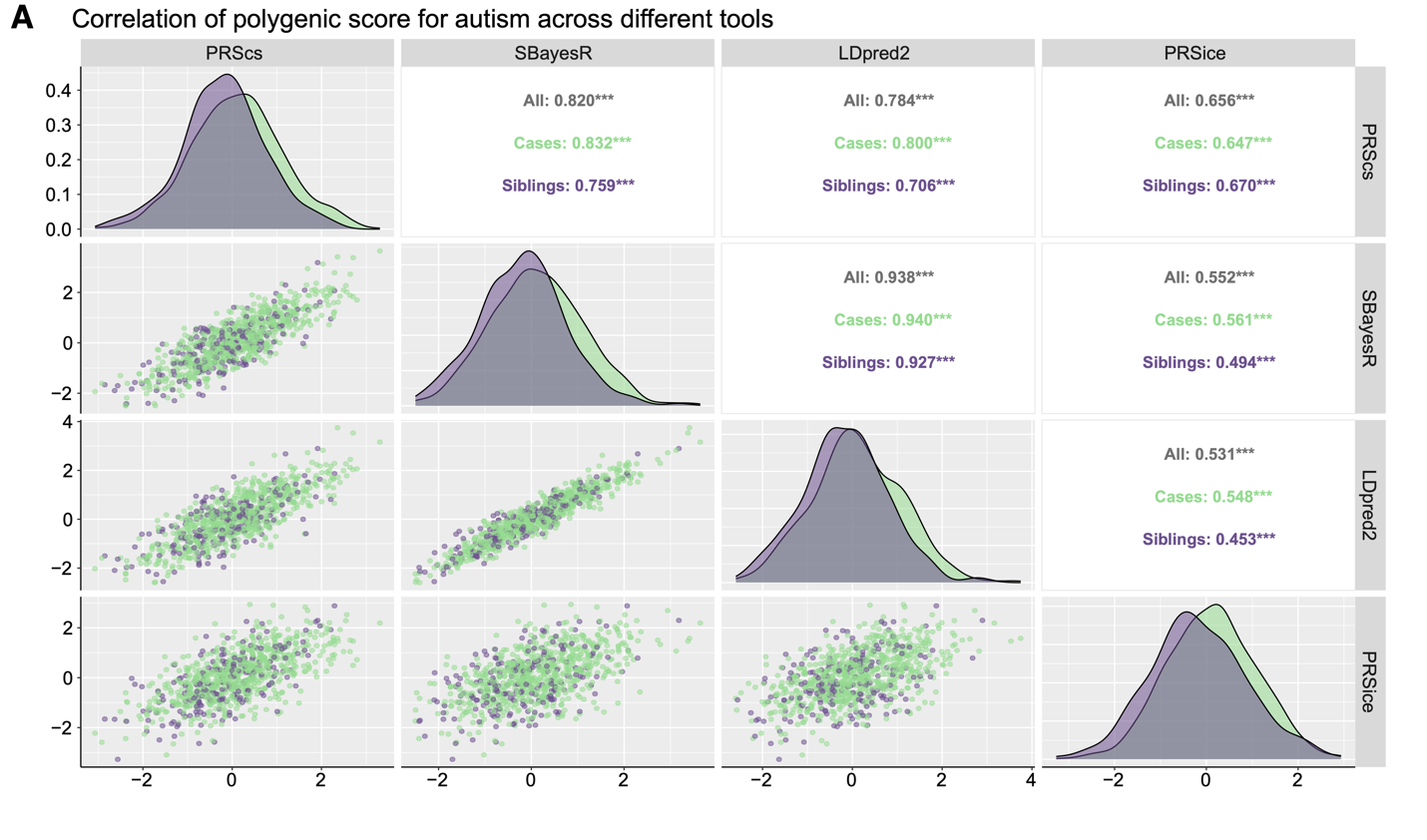
**

**Fig. S2| PS from different methodologies**

**A,** Correlation of PS across 4 different methodologies for calculating PS. R^2^ and *P*-values were computed from a linear regression. Autism status is represented by colors (green, autism cases; purple, non-autistic siblings) and the significance of *P* < 1.0x10^-12^ is denoted by ‘***’.


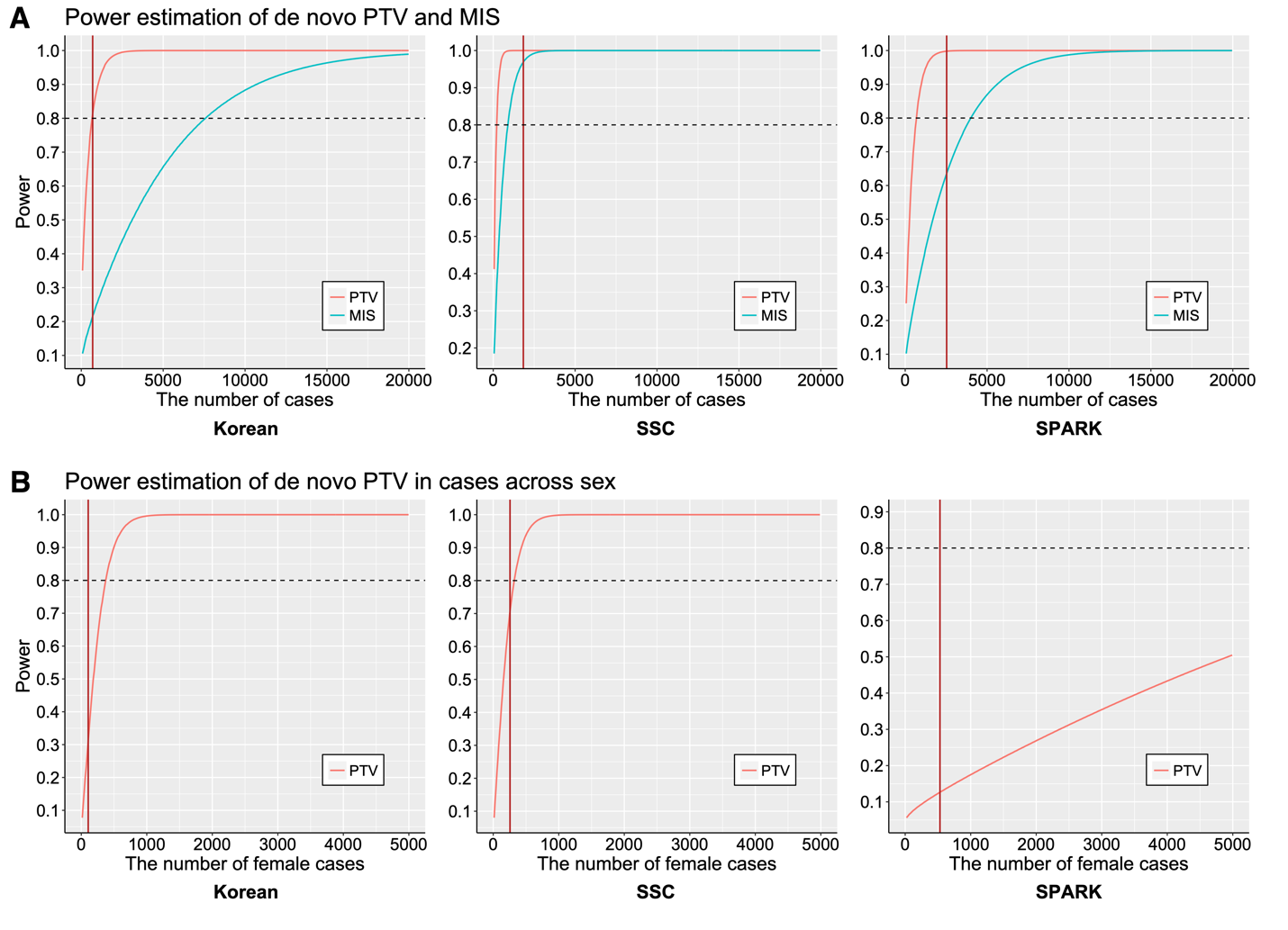


**Fig. S3| Power calculation of *de novo* burden test**

**A-B,** Power estimation for risk ratio (RR) in Korean, SSC, and SPARK cohorts; **A,** for *de novo* PTVs and MIS across individuals with autism and non-autistic siblings; **B,** for *de novo* PTVs in individuals across sex. The power of RR was computed by binom.power() function in R. The success probabilities under the null hypothesis are the ratio of individuals with autism out of total samples. The success probabilities under the alternative hypothesis are the ratio of DNVs. The number of independent trials is the sample size. Power estimation was iterated followed by the increase in sample size. X axis of the figure was calculated by multiplying the ratio of individuals with autism to the sample size. Red vertical lines display the total number of cases in the current datasets. Type of variant is represented by colors (orange pink, damaging PTV; blue green, damaging missense).

**
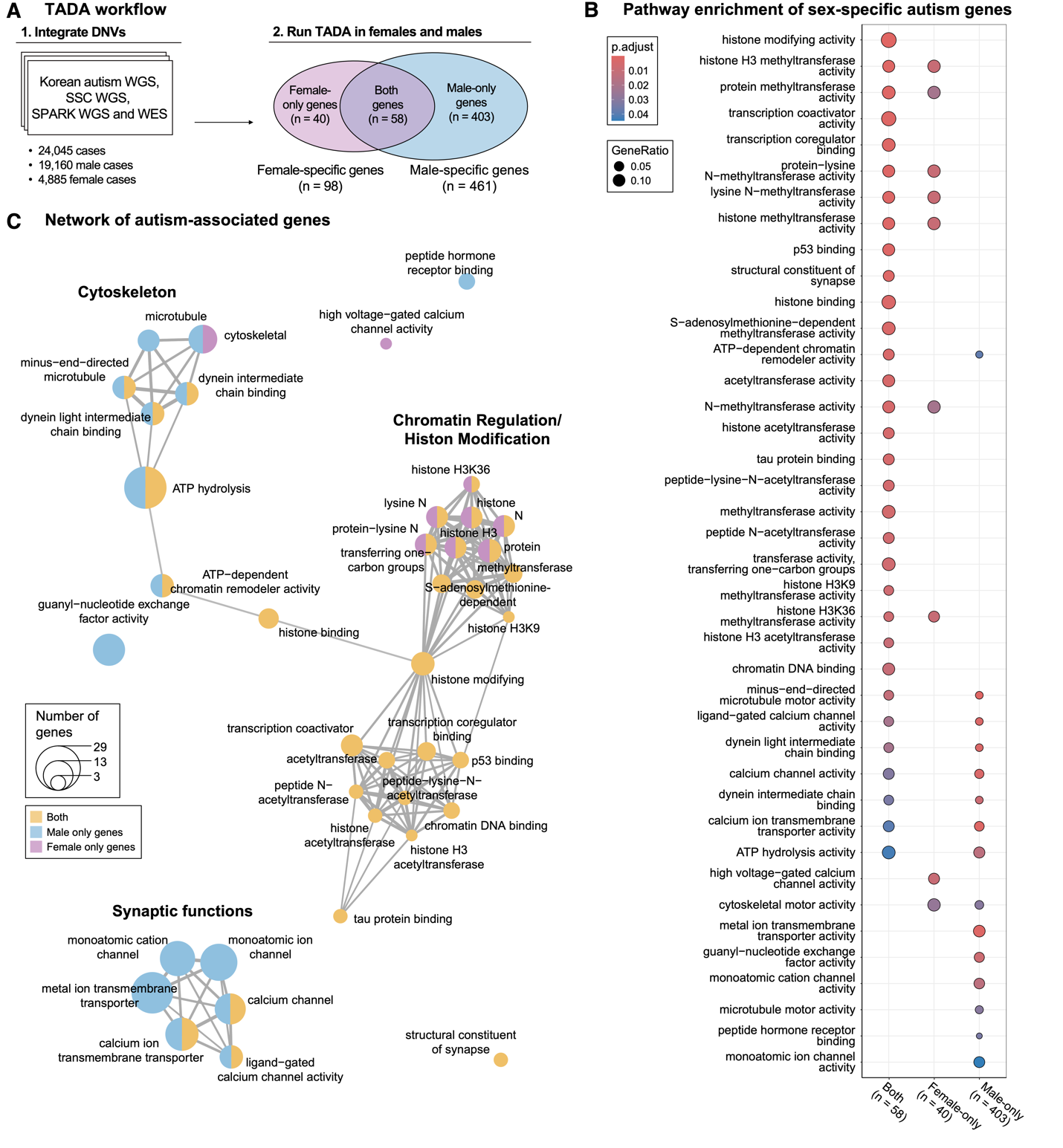
**

**Fig. S4|** **Sex-specific autism-associated genes**

**A,** TADA workflow for identification of sex-specific autism-associated genes.

**B,** Biological pathways enriched for TADA female genes, male genes, and both genes (overlap between female and male genes). Each row represents a different biological pathway, and the size of the circle in each column corresponds to the gene ratio involved in each pathway, colored by the adjusted p-value significance.

**C,** The network of enriched biological pathways of female-only genes, male-only genes and both genes. The number of genes involved is represented by size of circle and whether the pathway is enriched by female-only genes, male-only genes or both genes is represented by colors (pink, female-only genes; light blue, male-only genes; yellow, both genes).


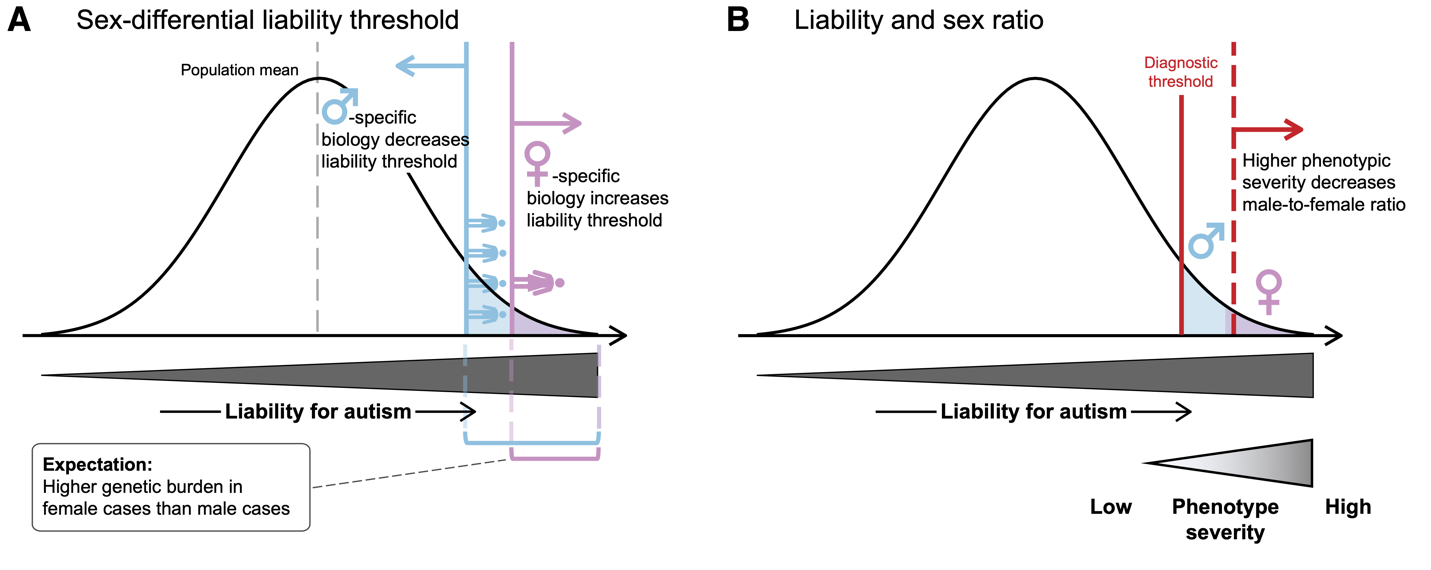


**Fig. S5|** **Sex-differential liability threshold model**

**A,** Sex-differential liability threshold for autism. Sexes are represented by colors (pink, female cases; light blue, male cases).

**B,** Relationship between sex-differential liability threshold and male-to-female sex ratio.


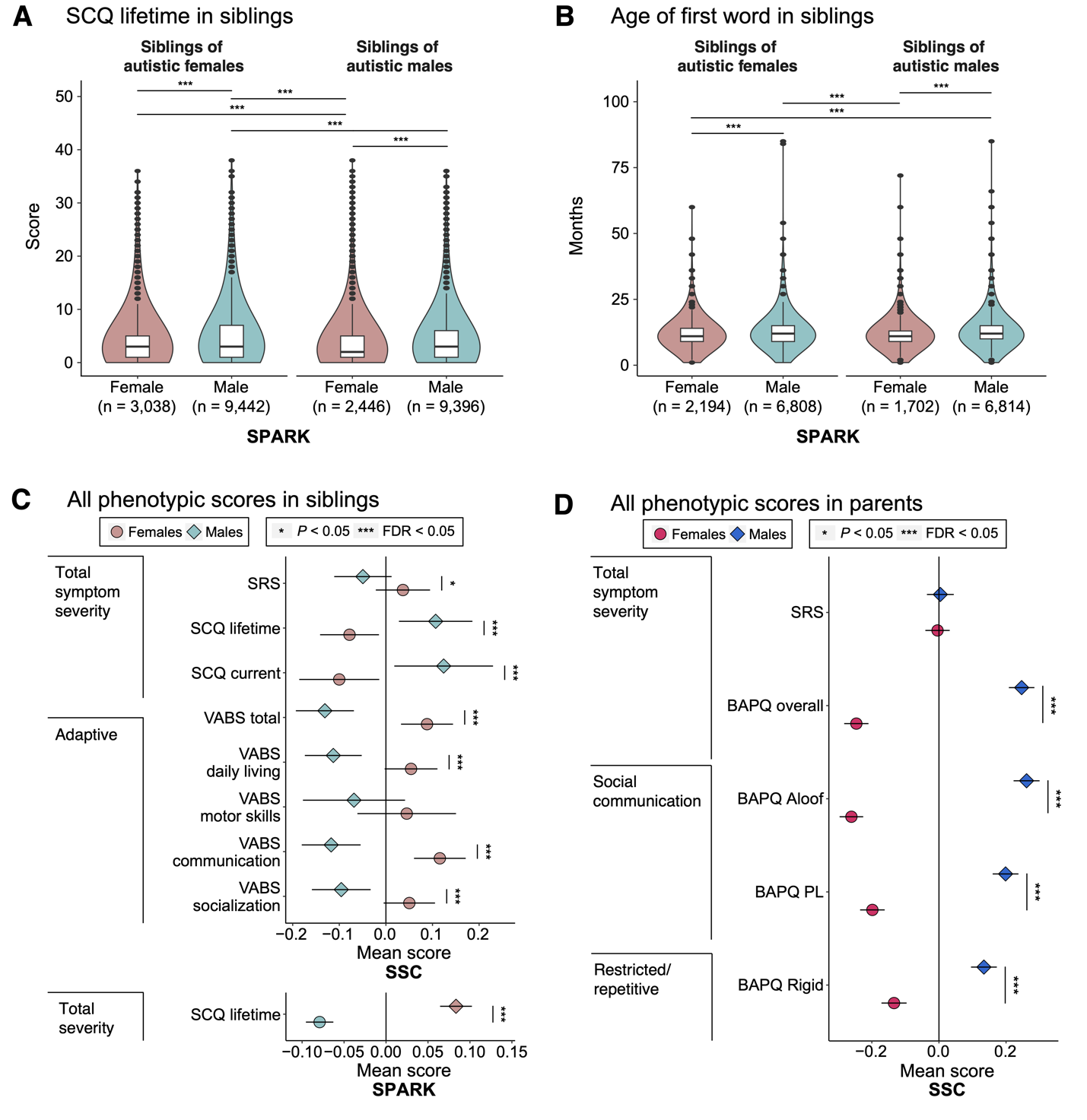


**Fig. S6|** **Sex differences of phenotypic scores in siblings and parents in the replication cohort**

**A-B,** Comparison of total symptom severity and developmental age across 4 groups of sibling-case sex pairs in siblings in SPARK. Phenotypic scores include **A,** SCQ lifetime (total symptom severity); **B,** age of first word (developmental age). Two-way ANOVA test, followed by Tukey’s multiple comparisons, was conducted and only adjusted *P*-values < 0.05 are displayed. Sex is represented by colors (dark pink, female siblings; blue-green, male siblings).

**C,** Comparison of z-transformed phenotypic scores including total symptom severity, social communication, restricted/repetitive behaviors, and cognitive/adaptive scores across sex in siblings in SSC, and SPARK. Two-sample t tests were used for contrasts. Points represent mean scores, and error bars represent the 95% CIs. Sex is represented by colors and shapes (blue-green circle, female siblings; dark pink diamond, male siblings) and the significance level is denoted by asterisk (‘***’, FDR < 0.05; ‘*’, *P* < 0.05).

**D,** Comparison of z-transformed total symptom severity scores across sex in parents in SSC. Two-sample t tests were used for contrasts. Points represent mean scores, and error bars represent the 95% CIs. Sex is represented by colors and shapes (red circle, mothers; blue diamond, fathers) and the significance level is denoted by asterisk (‘***’, FDR < 0.05; ‘*’, *P* < 0.05).


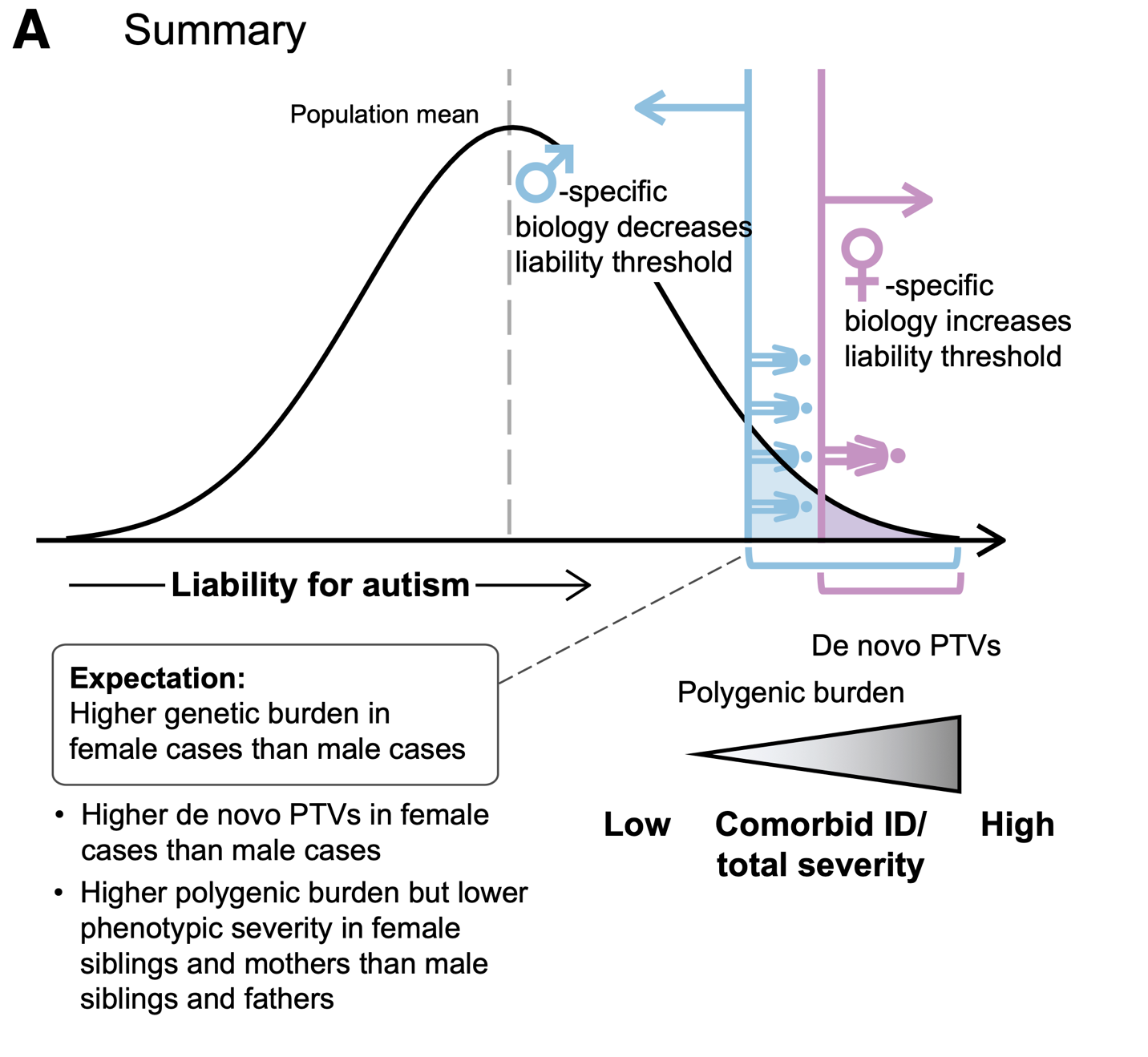
**Fig. S7|** **Key findings under the sex-differential liability threshold model**

**A,** Key findings in this study under the sex-differential liability threshold model. Sexes are represented by colors (pink, female cases; light blue, male cases).


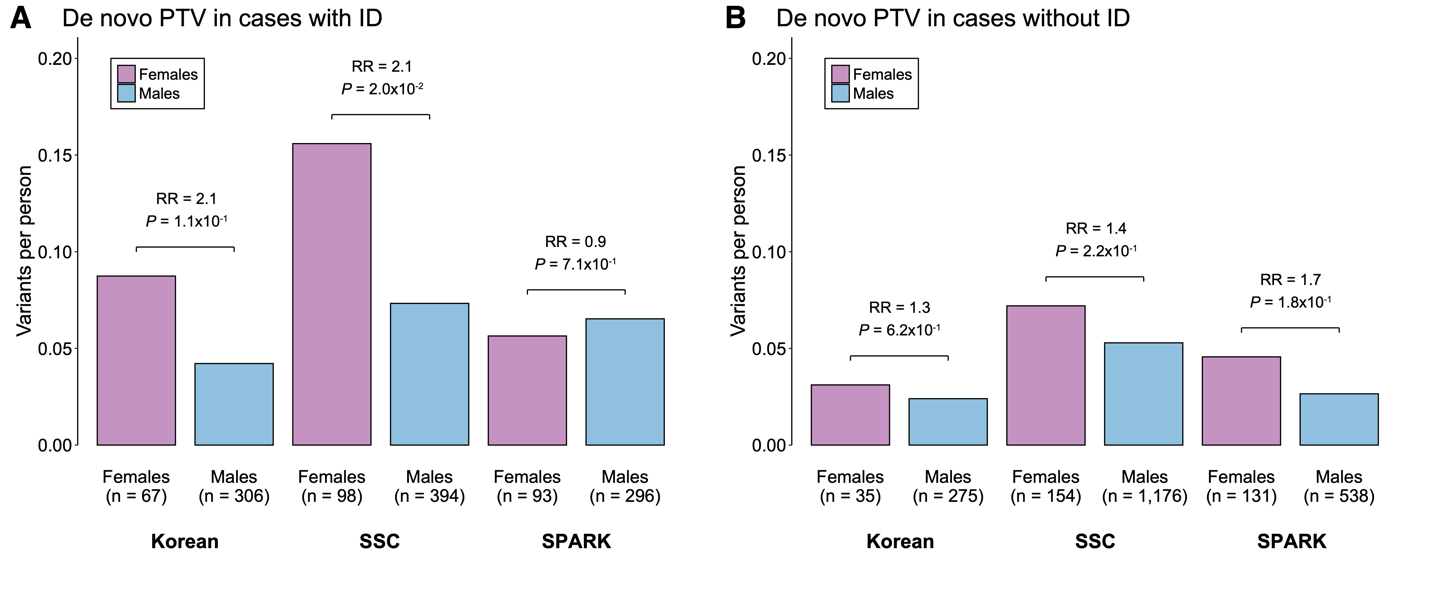


**Fig. S8|** **Effects of ID and total symptom severity on sex differences in *de novo* and polygenic burden**

**A-B,** Comparison of the *de novo* PTVs in constrained genes, adjusted for paternal age at birth across Korean autism, SSC, and SPARK cohorts between sex; **A,** among children with autism and ID; **B,** among children with autism and without ID. The y axis indicates the average number of variants. The *P*-values were computed by one-sided exact binomial test. Groups and sexes are represented by colors (green, autism cases; purple, non-autistic siblings; pink, female cases; light blue, male cases).
